# Peptide-Based Vaccine against Breast Cancer: Recent Advances and Prospects

**DOI:** 10.3390/ph16070923

**Published:** 2023-06-25

**Authors:** Muhammad Luqman Nordin, Ahmad Khusairi Azemi, Abu Hassan Nordin, Walid Nabgan, Pei Yuen Ng, Khatijah Yusoff, Nadiah Abu, Kue Peng Lim, Zainul Amiruddin Zakaria, Noraznawati Ismail, Fazren Azmi

**Affiliations:** 1 Centre for Drug Delivery Technology, Faculty of Pharmacy, Universiti Kebangsaan Malaysia (UKM) Kuala Lumpur Campus, Jalan Raja Muda Abdul Aziz, Kuala Lumpur 50300, Malaysia; luqman.n@umk.edu.my; 2 Department of Veterinary Clinical Studies, Faculty of Veterinary Medicine, Universiti Malaysia Kelantan (UMK), Pengkalan Chepa, Kota Bharu 16100, Kelantan, Malaysia; 3 Institute of Marine Biotechnology, Universiti Malaysia Terengganu, Kuala Terengganu 21030, Malaysia; madkucai89@gmail.com; 4 Faculty of Applied Sciences, Universiti Teknologi MARA (UiTM), Arau 02600, Malaysia; abuhassannordin@gmail.com; 5 Departament d’Enginyeria Química, Universitat Rovira I Virgili, Av. Països Catalans 26, 43007 Tarragona, Spain; wnabgan@gmail.com; 6 Drug and Herbal Research Centre, Faculty of Pharmacy, Universiti Kebangsaan Malaysia (UKM), Jalan Raja Muda Abdul Aziz, Kuala Lumpur 50300, Malaysia; pyng@ukm.edu.my; 7 National Institutes of Biotechnology, Malaysia Genome and Vaccine Institute, Jalan Bangi, Kajang 43000, Malaysia; kyusoff@upm.edu.my; 8 UKM Medical Molecular Biology Institute (UMBI), UKM Medical Centre, Jalan Ya’acob Latiff, Bandar Tun Razak, Cheras, Kuala Lumpur 56000, Malaysia; nadiah.abu@ppukm.ukm.edu.my; 9 Cancer Immunology & Immunotherapy Unit, Cancer Research Malaysia, No. 1 Jalan SS12/1A, Subang Jaya 47500, Malaysia; kuepeng.lim@cancerresearch.my; 10 Borneo Research on Algesia, Inflammation and Neurodegeneration (BRAIN) Group, Faculty of Medicine and Health Sciences, Universiti Malaysia Sabah, Jalan UMS, Kota Kinabalu 88400, Malaysia; zaz@ums.edu.my

**Keywords:** breast cancer, immunotherapy, peptide-based vaccine, nanoparticle, metastasis

## Abstract

Breast cancer is considered the second-leading cancer after lung cancer and is the most prevalent cancer among women globally. Currently, cancer immunotherapy via vaccine has gained great attention due to specific and targeted immune cell activity that creates a potent immune response, thus providing long-lasting protection against the disease. Despite peptides being very susceptible to enzymatic degradation and poor immunogenicity, they can be easily customized with selected epitopes to induce a specific immune response and particulate with carriers to improve their delivery and thus overcome their weaknesses. With advances in nanotechnology, the peptide-based vaccine could incorporate other components, thereby modulating the immune system response against breast cancer. Considering that peptide-based vaccines seem to show remarkably promising outcomes against cancer, this review focuses on and provides a specific view of peptide-based vaccines used against breast cancer. Here, we discuss the benefits associated with a peptide-based vaccine, which can be a mainstay in the prevention and recurrence of breast cancer. Additionally, we also report the results of recent trials as well as plausible prospects for nanotechnology against breast cancer.

## 1. Introduction

Among women worldwide, breast cancer is considered the most frequently occurring cancer. According to the World Health Organization (WHO), 2.1 million females have been diagnosed with breast cancer every year, which is responsible for approximately 15% of all cancer deaths [1]. Breast cancer is a cancer that develops from the epithelial cells of the mammary gland, duct, or lobules. Breast cancer occurrence also exists in males; however, it is relatively rare (around 1%) [2,3,4]. Although pathophysiological knowledge of breast cancer is minimal, certain established risk factors, such as genetic predisposition (family history), diet, and an unhealthy lifestyle, are unquestionably linked to the development of breast cancer [5,6,7,8]. To highlight, both genetic and environmental factors influence the diversity of breast cancer etiology [9,10].

It is widely accepted that BRCA 1 and BRCA 2 genes are responsible for repairing DNA dysregulation, alteration, and damage. These genes are predicted to be accountable for approximately 40–85% of the risk of hereditary breast cancer when they are mutated. BRCA 1 and BRCA 2 are located on chromosomes 17q and 13q12–13, enabling the inference of more distally mutated loci associated with mutations to affect their functional enhancers and promoters’ actions. Besides BRCA germline families, mutations in p53, PTEN, CHEK2, ATM, PALB2, RAD51C, and RAD51D have also been associated with breast cancer [11,12]. Additionally, mutations in BRCA genes can lead to the acquisition of a multi-drug resistant phenotype, subsequently contributing to a major limitation in clinical treatment for breast cancer [13,14,15].

The condition can worsen if the genes are inherited from one generation to another and become inheritable mutations. It can happen when epigenetically mutated cells accumulate and then further create a microenvironment that improves drug efflux, drug evasion, the anti-apoptotic pathway, and other escape mechanisms from the immune system. Epigenetic mutations, resulting mostly in DNA methylation patterns, histone acetylation, and phosphorylation, are known to have a profound effect on gene expression, resulting in the activation of tumor suppressor genes and leading to the emergence of cancer drug resistance [16]. When malignant cells continue to metastasize, they overly express immune checkpoint inhibition (CPI) signals, resulting in stimulation of inhibitory co-stimulatory molecules (PD1/PD-L1/LAG-3, CTLA4) and anti-apoptotic signaling pathways, causing tumor cells to deactivate immune activation and immune detection; hence, the tumor cells escape and progress to cancerous form [17]. Interactions of cancer in the tumor microenvironment can activate cellular components in the environment, including tumor-associated macrophages (TAM), cancer-associated fibroblasts (CAF), and mesenchymal cells, to protect cancer cells from being susceptible to drugs and promote drug resistance [18]. Some of the cancer biomarkers can also hinder tumor antigen expression, leading to the failure of the intended drug to penetrate cancer due to the unfavorable and diverse mutational landscape possessed by the cancer’s microenvironment, which additionally creates immunometabolism barriers [19].

**Figure 1:**
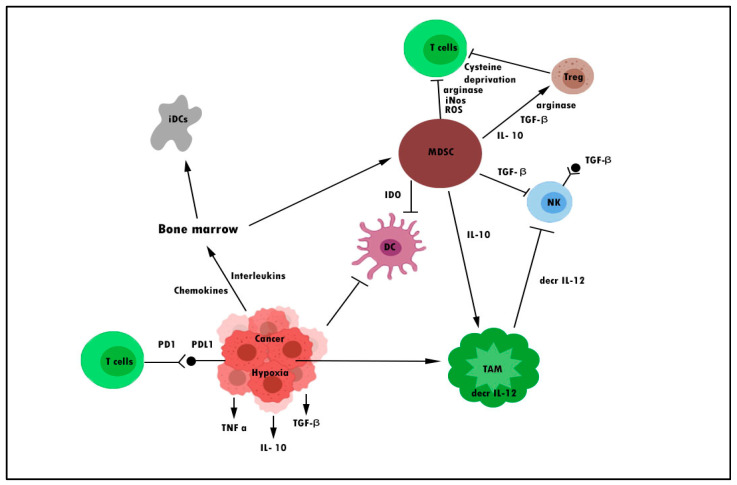
Mechanisms of cancer escaping pathways from the immune system. DC denotes dendritic cell, iDC denotes immature dendritic cell, MDSC denotes myeloid-derived suppressor cell, decr IL-12 denotes a decrease in interleukin-12, TAM denotes tumor-associated macrophage, and Treg denotes regulatory T cell.

Myeloid-derived suppressor cells (MDSCs) are one form of immune cell that plays a major role in tumor immunosuppression. These cells consist of immature monocytes and granulocytes released from the bone marrow into the blood during disease conditions, including cancer. Tumor-associated macrophages (TAMs) are another type of cell that functions similarly to MDSCs. The ability of MDSCs and TAMs to suppress the antitumor response is the subject of many recent studies [20,21]. MDSCs could suppress not only natural killer (NK) cells and dendritic cells (DCs) but also T cells. T cells were suppressed through the production of inducible nitric oxide (NO), nitric oxide species (iNOS), reactive oxygen species (ROS), arginine, and cysteine deprivation. Meanwhile, MDSC is able to synergize T regulatory cells (Treg) and TAMs and cause downregulation of IL-12 production by TAMs, which is an important cytokine involved in T cell and NK cell activation through membrane-bound TGF-β [22,23,24]. Figure 1 demonstrates some of the mechanisms of cancer cell evasion via hijacking the immune system.

The presence of tumor immunogenicity in the breast cancer microenvironment has necessitated the use of immunotherapy as a potential cancer treatment [25,26]. Immunotherapy can target specific cells that are involved in hijacking the immune cells; thus, it seems to be a good idea for the therapy’s success. Immunotherapy in the form of a vaccine functions by utilizing the patient’s immune system to identify and eradicate cancerous cells. Cancer cells produce chemokines, cytokines, and prostaglandins that attract diverse infiltrating immune cells consisting mainly of macrophages, neutrophils, and lymphocytes [27]. These infiltrating immune cells can stimulate tumor necrosis factor (TNF), IFN, matrix metalloproteinases, natural killer (NK) cells, and T cells, leading to the destruction of cancer cells. Most targeted therapies in recent years for cancer immunotherapy involve utilizing and targeting enough tumor-infiltrating T lymphocytes (TILs) and cytotoxic T cells (CTLs), which may correlate with the presence of antigen loads and suppress immune inhibitory signals responsible for local immunosuppression of the tumor microenvironment [28,29]. The discovery of breast cancer tumor-associated antigens (TAAs) or tumor-specific antigens (TSAs), which are expressed in breast cancer cells, has made it possible to develop a vaccine against breast cancer. Therefore, an understanding of the immune cell population may have significant consequences for the prevention of breast cancer, enhanced risk management strategies, and the control of breast carcinogenesis. 

In this review, we provide an overview of the recent advancements in the development of a peptide-based vaccine against breast cancer, with emphasis given to antigen selection and vaccine design. Additionally, breast cancer immunology and considerations for future directions for rational breast cancer vaccine design will be discussed.

## 2. Cancer Vaccines

The fundamental understanding of tumor immunology and its plausible mechanism of action has opened the route to employing the body’s immunity against cancer [30]. Immunotherapy in the form of a vaccine has great potential for breast cancer treatment over chemotherapy and endocrine therapies due to several issues, including relapse and drug resistance. Recent reports demonstrate that about 80% of treatment failures are due to metastases and drug resistance from several mechanisms of action, such as genetic mutation [13,14]. The management of advanced malignant breast cancer, with median overall survival ranging from 4 to 5 years for luminal-like tumors to 1 year for triple-negative disease, remains minimal and is considered short [31]. Until now, many scientists have tried to discover how to overcome therapeutic resistance because more than one mechanism may be responsible for oncogenesis. Even though there are several immunotherapy forms, including adoptive cell transfer, checkpoint blockage, and antibody-based drugs, vaccines are seemingly more tempting due to their wide safety profile and lifelong protection [32,33,34]. However, until now, no breast cancer vaccine has been authorized by the U.S. Food and Drug Administration (FDA) for either therapeutic or prophylactic purposes. The immune system in humans is incredibly complex. Even though, until now, no breast cancer vaccine has been authorized, many are still in clinical trials. It is only a matter of time. The tumor microenvironment (TME) in tumors remains one of the major hurdles in developing a therapy against breast cancer. Breast cancer is generally infiltrated by immune cells triggered by the cancer cells, which can create an immunosuppressive microenvironment that encourages tumor growth by inhibiting immune cells [35]. Besides TME, the following factors are thought to be responsible for the challenges of breast cancer vaccine development: (i) the stage of breast cancer; (ii) the choice of TAAs to target; and (iii) the vaccine’s low immunogenicity as a result of the antigen selected or as a result of the vaccine delivery platform used [36].

There is, however, increasing attention in clinical research that evaluates vaccines derived from the peptide. The rationale for this interest is based on the aberrant expression of proteins or antigens by breast cancer. With the discovery of breast cancer antigens, the peptide-based vaccine is becoming a potential alternative to conventional therapies, which are known to have serious drawbacks. The vaccines would modulate the immune system of the body to specifically attack cancer cells based on the recognition of tumor associate antigens (TAAs) or tumor-specific antigens (TSAs) on the surface of cancer. Interestingly, recognition of these antigens eventually allows the immune system to recognize these antigens, to have long-lasting immunity, and to solve the relapse issue after completion of the treatment. A robust, fundamental, and precise comprehension mechanism for the action of peptide vaccines is required to establish potent and effective cancer vaccines. 

## 3. Peptide-Based Vaccine and Key Regulator in Breast Cancer Immunogenicity

A peptide-based cancer vaccine is a short chain of amino acids that contain epitopes that are reactive to T cells. The major objective of peptide-based cancer vaccines is to induce the necessary host immune response to recognize and eliminate targeted cancer cells based on a defined set of TAAs and TSAs. The peptide-based vaccine follows the principle of immunotherapy, which modulates the immune system of the body to specifically attack cancer cells based on the recognition of aberrant expression of tumor antigens or proteins in the cancer cell. Interestingly, recognition of these antigens eventually allows the immune system to recognize these antigens, to have long-lasting immunity, and to solve the relapse issue after completion of the treatment. 

**Figure 2:**
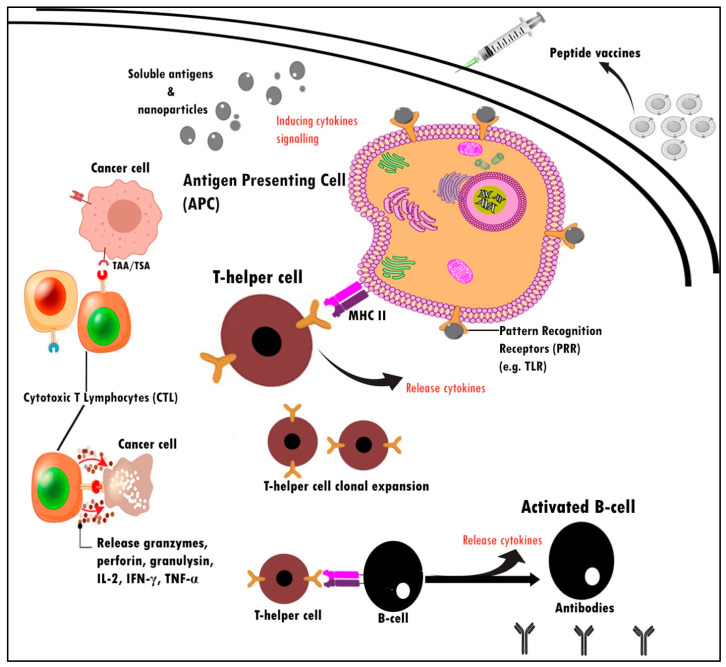
Schematic illustration mechanism of action for peptide vaccines. These immunological events are essential for enhancing the cell-mediated and humoral immune response against peptide vaccines. PRR is expressed by APCs and acts by recognizing and binding with antigens.

Administration of a peptide vaccine functionalized CD8+ cytotoxic T lymphocytes (CTL) cells to attack tumor cells through the release of granozymes, granulysin, perforin, and Fas ligand (FasL) through Fas death receptor binding to cancer cells for apoptosis to occur (Figure 2). Cytokine release helps with lymphocyte migration, B cell development, T cell activation, and expansion. After activation, CD4+ T cells further differentiate to develop dominant anticancer pathways and responses. In order to modulate tumor-specific immune responses and inhibit proliferation in the body, significant interventions have been rendered to identify tumor-expressed antigen cells or those recognized as tumor-associated antigens (TAAs) utilizing T cells [37,38]. Identified as an example of HER2 antigen in breast cancer and transformed into a vaccine component capable of triggering a specific and systemic immune response that may contribute to the suppression, removal, and destruction of cancer in body tissues [39]. When TAAs are found in the body from cancer cells, specific fragments of cancer proteins are expressed on the cell surface and then attached to the MHC 1 complex [40]. It would ultimately be recognized by NK cells and CD8+ cells. The dissimilarity between endogenous and exogenous peptides is crucial for a functional immune response.

T-cell responses are specific and triggered after peptides have been taken up and processed by antigen presentation cells (APCs), which then transit to lymph nodes and expose the antigen on their cell surface as foreign molecules through MHC-I and II [41,42]. Prolonged activation of T cells leads to further differentiation into CD8+ cytotoxic and CD4+ helper T cells through the CD40-CD40L pathway. Studies have shown that CD40-CD40L associations are capable of inducing humoral and cellular thymus-dependent (TD) reactions [43]. When APCs are triggered through CD40-CD40L, it is found that ligation of CD40-CD40L to APCs, particularly dendritic cells, is capable of generating cytotoxic CD8+ T cells [43]. This may grant CD8+ T cells the potential to modulate antigen-specific immune responses similar to the CD4+ T cell response, which is often correlated with CD40L expression [44,45]. The use of TAA-related peptides such as GP2 tends to be an efficient way to stimulate CD8+ and CD4+ production against cancer cells.

Peptide-based vaccines offer several advantages over other types of cancer vaccines. Peptide-based vaccines can be easily customized with minimal epitopes while still being able to induce desired immune responses safely. Peptides are manufactured almost entirely using synthetic chemical approaches. Therefore, peptide antigens can be completely and specifically identified as chemical entities. Hence, all issues associated with the biological contamination of antigens are effectively eliminated.

Despite its benefits, peptide-based vaccination also exhibits several drawbacks, including a limited half-life, an insufficient immunogenic response, being easily degradable, and low bioavailability. However, due to the heterogeneity between solid tumors and the external microenvironment, the efficacy of the immune response in solid tumors is not as anticipated [46]. Therefore, by modifying the delivery system of peptides, such as nanocarriers that have a protective layer and are bound to the TAAs, it is then possible to prevent the degradation of these proteases and improve the association between peptide vaccines and cancer. Recent studies and many clinical studies have uncovered the potential use of peptide-based vaccines as immunotherapeutic agents that may weaken or break the immune tolerance of cancer patients. However, several modifications need to be made to the peptide vaccine to reach ideal potency. 

The clinical efficacy of peptides can be easily enhanced by covalently conjugating or linking chemically with specific immunostimulatory molecules at specific positions within the peptide sequence. The use of only minimal antigens is capable of triggering humoral and cell-mediated immunities. Peptides applied in breast cancer vaccines (Table 1) and selected breast cancer peptides in clinical trials (Table 2) provide convincing evidence that peptide-based vaccines are a viable strategy for the treatment of breast cancer. A peptide-based vaccine depends on mobilizing cytotoxic CD8+ T cells and NK cells to kill the cancerous cells. To stimulate a tumor-specific immune response, TAAs or TSAs must be presented to the APC and make the immune system of the host recognize them. Several TAAs are mainly identified as immune targets for a vaccine against breast cancer. This includes HER2, MUC-1, EphA, Survivin, SART3, CEA, p53, and WT1 [47,48,49]. These antigens have provided convincing evidence as immune targets in preclinical and clinical studies and warrant further research.

Present clinical studies have demonstrated the therapeutic value of peptide-based vaccines to reduce cancer recurrence and enhance overall patient survival [50,51]. NeuVax™ (NCT01479244) is the most mature level of production for a peptide-derived breast cancer vaccine. It was in Phase III clinical trials and was initiated by the US National Cancer Institute in 2011 [52]. Targeting precise TAAs is vital to induce successful T-cell differentiation and alarm signals for tumor destruction mechanisms. In order to obtain a TAA-specific T cell response and upregulate stimulatory signals, APC needs to be provided with enough TAAs and be in a mature state. Otherwise, antigens may not promote oncogenesis and trigger T helper-cell clonal expansion. This is essential because CTLs are a significant cell type responsible for killing cancer cells. Various peptide breast cancer antigens that may trigger an immune response in patients have been identified and used as targets for breast cancer vaccines. However, the reduced immunogenicity of these peptide cancer antigens and cancer immune evasion mechanisms makes the development of breast cancer vaccines challenging. This condition necessitated the need to build an efficient vaccine delivery system with powerful immunostimulatory properties to promote APC activation, thus eliciting a strong T cell response and weakening and breaking the immunotolerance of cancer antigens in the tumor microenvironment.

Noteworthy, breast cancer is known to be a complicated, non-infectious, and immunogenic disease with the ability to alter the tumor microenvironment, making it resistant to treatment. In this case, manipulating the body’s immunological reaction to cancer will provide some merit for potential cancer vaccine research. Peptide detection by Human Leukocyte Antigens (HLA), which are gene coding for the Major Histocompatibility Complex (MHC), has also become a challenge because each HLA has its own subtypes, and each subtype can be specific to TAA proteins. For instance, GP2 is a Class I peptide MHC that is restricted to HLA-A2 and/or HLA-A3 [51]. Nevertheless, the function of HLA in determining the prognosis and effectiveness of peptide vaccines is still uncertain [53,54,55]. Research published by Jackson et al. [53] showed that HLA-A2 expression did not substantially correlate with the prognosis of women with breast cancer. This finding is essential for the selection of candidates for the HLA-A2 breast cancer vaccine. A peptide can target important ligands in breast cancer regulatory pathways, including chemokines, Y-box binding protein-1 (YB-1), Sin3, TNF 1-related ligand-inducing apoptosis (TRAIL), and FasL [56].

## 4. Identified Tumor-Associated Antigens in Peptides Vaccine Development for Breast Cancer

### 4.1. HER2

HER2, also known as ERBB2, NEU, and CD34, is a human epidermal growth factor receptor 2 and a component of transmembrane glycoprotein that is overexpressed in approximately 20–30% of primary breast carcinomas [47,57] for tyrosine kinase activity. The HER2/neu cell surface receptor is the most frequently targeted TAA; thus, the HER2-derived peptide vaccine has shown excellent potential in developing breast cancer vaccines. Upon dimerization of the antigen receptor, the numerous intracellular signaling pathways are activated by transphosphorylation, which mediates cell proliferation and differentiation. However, when inappropriate activation happens, it contributes to the production of many malignancies [58]. Slamon et al. [59] first discovered the function of HER2 as a marker with a prognosis value for treating breast cancer in 1987. It has been confirmed and proved by several scholars [60,61,62]. Studies conducted by Clynes and colleagues reported monoclonal antibodies targeting HER2 to provide clinical benefits against HER2 overexpressing breast carcinomas [63,64]. To date, the application of HER2 as a therapeutic marker and predictor in invasive breast carcinomas has been commonly utilized and continues to develop. 

To sum up, HER2/neu is a well-known therapeutic target that is a hallmark of HER2-positive breast cancer. With HER2-targeted vaccinations, targeting HER2 appears to be a reasonable strategy for the dysregulation of numerous signaling cascades that promote oncogenesis. It has been extensively used with a GM-CSF adjuvant. They offer little or no risk and the chance of producing a memory antibody against the same disease. Ex vivo expansion of cellular immunity, including activation of CD8+ CTL against breast carcinoma, will be enabled by the production of anti-HER2 immunization. Vaccinated patients showed high levels of CD8+ and mediated delayed-type hypersensitivity reactions [64]. Established by Mansourian et al. [65], p5 peptide encapsulated with liposomes co-administrated with CpG-ODN has been shown to decrease tumor size and, at the same time, improve animal survival period in mice of the breast cancer model. This was confirmed by Farzad et al.’s study [57], which displayed another peptide, the P435 HER2-derived peptide, conjugated to liposomes capable of inducing CTL responses, therefore improving prognosis in the TUBO murine breast cancer model. Another study revealed that Nelipepimut-S (E75) was a nine-amino acid peptide extracted from the HER2 protein capable of increasing the patient’s survival rate. Research from recent clinical trials has demonstrated positive effects of HER2-specific vaccinations that, when paired with chemo-drugs, could synergistically inhibit the recurrence of breast cancer, creating robust immunity and sustaining elevated CTL rates. One of the hurdles to the HER2-based vaccine is against TNBC subtypes due to the lack of HER-2 (ERBB2), progesterone, and estrogen receptors. Costa and colleagues suggested that combinations of HER2-based vaccines with pembrolizumab or nivolumab (immune checkpoint inhibitor antibodies) merit promising outcomes [66]. Combinations with other therapies might produce synergistic effects and resensitize other cancer cell death programs.

### 4.2. MUC-1

Transmembrane mucin-like glycoprotein Mucin 1 (MUC-1) is a type of glycoprotein comprising a single polypeptide chain with multiple oligosaccharide side chains with oxygen linkages to serine, proline, and threonine residues [67] frequently overexpressed in glandular and epithelial mammary, lung, and colon cancers [68]. MUC-1 overexpression can be used as a marker for cancer that suggests that the cancer is progressing [49].

Several studies have demonstrated that targeting MUC-1 was a successful option for the cancer vaccine because it is broadly dispersed in all tumors and cancers, including stem cell cancer [69]. Covalently bound to the TLR agonist, the completely synthetic glycosylated MUC-1 peptide vaccine exhibited good humoral and cellular immune responses [70]. The pioneered MUC-1 peptide vaccine study was performed in 1995 against patients with breast carcinoma. The majority of patients reacted to the medication, and no toxicity was found [71]. The extension of the research was carried out up until the Phase III clinical trial. A pilot Phase III analysis of 31 early Stage II breast cancer patients utilizing oxidized mannan-MUC-1 immunotherapy found that MUC1 immunotherapy is effective [72]. The MUC-1 peptide vaccine candidates have demonstrated an improved survival rate. MUC-1 is proposed as a potential biomarker to be targeted in breast cancer therapy because patients would typically overexpress the MUC-1 biomarker (approximately 90%) for immune system detection. Antibodies against MUC-1 can efficiently cause CTL and TLR. Hence, reasonable disease regulation is accomplished as patients produce strong antibody titers of MUC1 IgG. In clinical studies on women with Stage I and Stage II breast cancer, MUC-1 IgG and IgM antibodies were tested and assessed for their association with disease-specific survival [49,73]. MUC1 is a possible antigen to be utilized as a site-specific target for the deployment of therapeutic agents as a vaccine against MUC1 for breast carcinoma. Recent studies have shown that MUC1 can induce antigen-specific cellular and humoral responses not only to trigger MUC1-specific CD4+ and CD8+ T-cells but also to generate antibodies [69].

### 4.3. EphA

EphA is a type of transmembrane glycoprotein with tyrosine kinase (RTK) receptors on the surface that play a significant role as tumor-specific cell-surface receptors for drug-targeting sites. It is the largest group among tyrosine kinase receptor families, and among them, EphA2 is commonly overexpressed in breast cancer. The activation and overexpression of EphA2 frequently lead to its ligand-independent oncogenic and angiogenesis activation, which are triggered by dwindled contact with the ligand, ephrin-A (EphA2). Loss of the ligated EphA2 receptor decreases the intrinsic tumor-suppressive signaling pathways, accompanied by downregulation of the PI3K/Akt and the ERK pathways, thus decreasing the tumor volume and size. 

As a therapeutic target, EphA2 receptors remain an essential marker. Overexpression of EphA2 receptors has been correlated with low survival in all patients with breast cancer subtypes due to the EphA2 activity that enhances tumorigenesis and the progression of metastases [74]. A monoclonal antibody (mAb EA5) has been studied to suppress EphA2 receptors in ER-positive breast cancer and to minimize cancer invasiveness [75]. The outcome was promising, and the study proceeded in the presence of tamoxifen. Furthermore, the monoclonal antibody EPhA2 can specifically target antigens and suppress the development of breast cancer cells and tumorigenesis.

YSA and SWL are peptide-based EpHAs that target EpHA2 receptors on the surface of tumor cells. Scarberry et al. [76] reported the success of using a magnetic CoFe_2_O_4_ nanoparticle-YSA peptide conjugate to extract ovarian cells from blood and fluid in mice. Even though the EpHA antigens are overexpressed in blood cancer and tumor cells, further exploration with regard to EphA as a peptide-based vaccine is very limited. Perhaps an EpHA-based vaccine does not elicit a potent immune response to eliminate various classifications of breast cancer. This may be triggered by cross-reactions between drugs and other proteins or by incomplete subcellular internalization of antibody-drug conjugates (ADCs). Thus, in order to address this issue, Salem et al. [77] proposed peptide-based targeting drugs that were less harmful but efficient and inexpensive. The aim of breast cancer therapy may be to merge EphA2 expression with carcinogenesis. Strategies focused on EphA2 targeting have been groundbreaking developments in therapeutic discovery. The targeted drug, for example, trastuzumab, is yet to be used; the concern of cardiotoxicity persists. Immunotherapy, similar to a cancer vaccination, tends to be an effective solution to treating metastatic breast carcinoma.

### 4.4. Survivin

Survivin, a 16.5 kDa intracellular acidic protein of 142 amino acids encoded by the BIRC5 gene, is a multifunctional protein that belongs to the smallest member of the inhibitory apoptosis protein family (IAPs). It regulates cell cycle progression through inhibition of the apoptosis pathway [78,79,80,81]. A high level of survivin expression is significantly associated with breast, urothelial, and colorectal cancer invasiveness and its low prognosis [82]. Survivin is undetectable in healthy tissue, indicating that it is exclusively presented as a biomarker when there is tumor transformation and acts as a transcriptome that is expressed in breast cancer. It seems to play a role in the antiapoptotic function of a protein, preventing the cell program from happening. A study by Ryan et al. [81] showed that a high level of survivin expression patterns is often associated with HER2/neu positive breast cancer and correlates with the prognosis.

This was confirmed by Lyu et al. [48], who reported that dysregulation of survivin was found in HER2/neu breast cancer, and survivin was identified as a desirable therapeutic target for blocking its IAP functions. A gene and immunotherapy named sepantronium bromide (YM155) have been developed to block survival. They provided a positive outcome for in vivo research by lowering the expression of survivin, raising the regression of the tumor, and prolonging the life of the mouse. However, YM155 failed to demonstrate an improvement in treatment response in metastatic non-small-cell lung cancer patients (NSCLC). This failure is probably due to the presence of multiple pathways linking survivin with other regulated proteins, making it more complicated [80,81].

Another research performed by Tanaka et al. [82] found that cytoplasm-responsive nanocarriers conjugated with a functional cell-penetrating peptide could facilitate the delivery of anti-vascular endothelial growth factor siRNA (siVEGF) complexes to tumor tissues after systemic injection and could elicit a potent anti-tumor effect. In a study from Rodel et al. [83], survivin as an antigen vaccine conferred peptide-specific CTL induction of urothelial cancers in patients without significant adverse reactions. On the other hand, the latest research indicated that the presence of the survivin antigen in breast carcinoma revealed a connection between expression and therapeutic outcomes [84].

### 4.5. SART3

SART3 is a tumor rejection antigen consisting of 3806 bp of nucleotides encoded by a 140-kilodalton (kDa) protein expressed in the cytosol of most of the cell proliferation and has been shown during gene transcription and mRNA synthesis of cancer cells. Similar to survivin, the SART3 oncogene is absent in normal tissues except for the fetal liver and testicles [85]. This antigen exhibits strong binding with HLA-A24-restricted CTL epitopes and may be useful for specific immunotherapy.

A Phase 1 clinical trial was recorded by Miyagi et al. [86] utilizing the SART3 peptide vaccine in colorectal cancer patients. The findings revealed a significant induction of cellular immune responses in 7 out of 11 patients. However, no explicit activation of the humoral immune response (IgG or IgE) has been recorded for peptides. SART3 led to the regulation of pro-inflammatory cytokine expression and the association of the degree of expression with malignancies and the prognosis for patients with breast cancer [87,88]. Given its positive outcome against SART antigen-expressed cancer, no clinical trials of SART-associated breast cancer vaccine goals have been reported.

### 4.6. CEA

CEA is a 180-kDa glycoprotein widely recognized as an oncofetal antigen that is found in numerous cancers, including colorectal, breast, gastric, pancreatic, and non-small cell lung cancers. CEA is one of the earliest tumor markers used to identify and anticipate the recurrence of tumors following surgical resection [89]. Its overexpression leads to the progression of the tumor. High secretions of CEA from cancer cells in the blood serum and over-expressed CEA on the surface of tumor cells make it accessible for use as a selective marker for cancer immunotherapy. CEA has been used as the foundation for numerous cancer vaccines, including DNA-based vaccines, dendritic cell-based vaccines, recombinant vector-based vaccines, protein-based vaccines, and anti-idiotype antibody vaccines, with the potential to induce both humoral and cell-mediated immunity that contributes to the killing of cancer cells [90]. Ojima et al. [91] demonstrated that genetically modified dendritic cells that express CEA administered simultaneously with interleukin 12 (IL-12), GM-CSF enhanced the therapeutic effects in CEA transgenic mice through the improvement of CEA-specific T-cell responses. Interestingly, the vaccination therapy eliminated colon cancer up to 2 × 10^3^ mm^3^ sizes. Furthermore, no detrimental results were found after the experiment. The research performed by Gulley et al. [92] found that 9 out of 16 patients diagnosed with recombinant CEA-MUC-1-TRICOM poxviral-based robust tumor vaccines had an increase in both CD8+ and CD4+ immune responses. To sum up, targeting CEA could be a successful vaccination technique for the clinical application of peptide vaccines to achieve a positive antitumor response.

### 4.7. p53

p53 is a tumor suppressor protein that plays a vital role in regulating genomic stability by controlling the cell cycle and inducing apoptosis when cell damage is beyond repair. p53 mutations occur in about 18–25% of primary breast cancer, rendering them potential biomarkers for cancer immunotherapy. Missense mutations within the p53 gene could potentially cause the accumulation of mutant proteins within the cell nucleus through posttranscriptional modification. The prognosis value of the patient appeared to be associated with the p53 level. Approximately 80% of TNBC patients have been identified with high p53 gene levels, and so far, no immunotherapeutic medication scientifically used for TNBC has been proven to develop a peptide-derived vaccine [93]. Many 2-sulfonyl pyrimidine compounds, such as PK11007 and PK11000, are successful in killing cancer cells by explicitly attacking mutant P53 thiol groups, thereby reducing oxidative stress levels (e.g., ROS) and eventually retaining a redox state [94,95].

PRIMA-1MET (APR-246) has been clinically studied in a Phase I clinical trial and is currently undergoing more clinical review. It inhibits cancer cell growth by targeting mutant p53 and inactivating it in triple-negative breast cancer (TNBC) [96]. In the Phase I/II clinical trial, ten colorectal cancer patients were vaccinated with p53-derived synthetic long peptides (SLPs). Rapid p53-specific T-cell responses were observed in blood samples obtained six months after the last vaccine [97]. Although the theory suggests that SLP would activate a high level of T cells in vaccinated patients, the clinical findings have unfortunately not been compatible. Perhaps targeting p53 alone is not enough to eliminate breast cancer cells. Thus, multiple peptides that target multiple antigens while stimulating multi-antigenic immune responses tend to be the right approach to improving the immunogenicity and clinical efficacy of p53-directed immunotherapies [98,99]. 

### 4.8. WT1

Wilms’ tumor 1 (WT1) is a gene located at chromosome 11p13, initially discovered in childhood kidney cancers and overexpressed in other hematological malignancies (leukemia) and solid cancers, including breast cancer, ovarian cancer, pancreatic cancer, renal cancer, endometrial carcinoma, and glioblastoma [100,101,102]. Additionally, WT1 expression has been identified and used as a potent transcriptional regulator and marker for myelodysplastic syndromes (MDS), acute myeloid leukemia (AML), and solid tumors, including breast carcinoma. This information indicates that WT1 can be a targeted antigen for cancer immunotherapy, and reducing its level shows inhibition of tumor progression [97]. Coosemans et al. [102] proved that 36 patients with endometrial cancers showed overexpression of WT1 in endometrial cells. This research is in conjunction with other reliable cancer reports [103,104,105]. Due to the fact that immunization principles for inducing an immune response are more or less identical in different peptide protocols, WT1 immunotherapy, fortunately, may provide diagnostic tools and prognostic markers for all solid cancers.

## 5. Strategies to Improve the Immunogenicity of Peptide-Based Breast Cancer Vaccines

Since peptides are not so immunogenic, few strategies for improving the effectiveness of peptide-based vaccines have been developed. The administration of immunostimulant agents (adjuvants), either mixed or chemically conjugated to the peptides, helps improve the body’s immunity against peptide antigens derived from the tumor. A rationale for incorporating immunostimulatory within the vaccine designs is to develop the synchronous activation of APCs (especially dendritic and macrophage cells) and foster T-cell responses without jeopardizing the quality and safety of the vaccine formulation [106]. Various types of vaccine-adjuvant formulations used in clinical trials are shown in Table 2. 

### 5.1. Multi-Epitope Peptide Vaccine Antigens

A key step in designing peptide-based vaccines is the choice of an epitope that is capable of stimulating robust, longer-lasting, targeted both cellular and humoral (or either one) immunity against the desired pathogen. Therefore, it is important to initially recognize appropriate peptide epitopes on the protein of interest. The selection of the epitope must also take into account the plausible hypersensitivity reaction that arises in correlation with certain antigens. Peptide vaccine development can be presented to the immune system in multi-antigenic forms, adopting the nature of pathogen properties. Ghaffari-Nazari et al. [107] reported that peptides containing cytotoxic T lymphocyte (CTL) epitopes elicit robust protective immunity against tumors. This was supported by Zamani and others, who demonstrated that CTL and T-cell epitopes derived from TAAs simultaneously stimulate the CD8+ cytotoxic and CD4+ T-cell responses. The pan-DR-biding epitope (PADRE) is an example of a T helper epitope that activates CD4+ T cells and has proven safe and well tolerated [104]. 

Studies conducted by Wu et al. [108] in mice using an E7 peptide-based vaccine and PADRE as an adjuvant showed stronger CTL responses compared to those without the T helper epitope. B-cell and CTL epitopes can be incorporated to generate specific and robust immune responses involving humoral and cellular immune responses. Each of the epitopes can stimulate immune responses. Therefore, combining multiple epitopes in a single system could provide synergism, increase IFN- production, and increase peptide affinity to MHC molecules, thus enhancing CD4+ and CD8+ T cell generation [109,110,111] because each epitope has its ability to trigger an immune response. Another way to enhance immunogenicity is by using long peptides. The long peptide sequences (30 mers) are believed to be more efficient in generating effector T cells due to their extra length, thereby requiring only professional APCs that can process and present MCH molecules [112]. In addition, extra length may provide a place for tertiary structure, thus making the peptide not easily degradable by serum peptidases and tissue [113]. These make peptides suitable to be used in tandem with bioengineering applications and vaccine design, which significantly improves their effectiveness without jeopardizing the safety or quality of the peptide [114,115,116].

Due to this reason, the peptide-based vaccine is now gaining a great deal of scientific attention and has stepped significantly forward in the field of cancer immunotherapy. Despite using protein, peptide within the protein is more feasible with a distinctive individual epitope that can induce protective immunity. Multiple immunogenic epitopes, for example, containing T and B cell epitopes, can be linked covalently to form stable and strong linear complexes of peptide sequences, thus providing the platform necessary for immune cell recognition. The vaccines destroying cancer cells depend on unique peptide antigens obtained from the TAAs and TSAs and enable the immune system, especially cytotoxic T lymphocytes (CTLs), of the host to recognize them. The overexpression of TAAs and TSAs on the tumor surface increases the exposure of tumor cells to targeted therapeutics, and interestingly, the receptor-mediated tumor-targeting ligands are a type of protein. These ligands allow targeted delivery of peptides of interest to the tumor site either by direct coupling or through a carrier delivery system such as liposomes, micelles, or nanoparticles, thus triggering the specific tumor immune response.

### 5.2. Immunostimulatory Adjuvants

#### 5.2.1. Toll-Like Receptors (TLRs) Ligands Based

TLRs belong to a class of pattern recognition receptors (PRRs) that are located at the surface or intracellular compartments of endosomal and cytoplasmic membranes, and they bind with the antigens before further creating intracellular signaling pathways that evoke immune system responses. Generally, TLRs resemble similar structural features of pathogen-associated molecular patterns (PAMPs) and have been easily recognized by PRRs. The ligand binding between TLR and antigen would activate the transcription factors nuclear factor kappa B (NF-B), interferon, and the release of cytokines such as IL6, IL1, IL8, IL12, TNF, and other molecules such as CD40, CD80, and CD86, which would lead to the recruitment of immune cells and, subsequently, induce killing mechanisms in cancer cells [117]. Examples of types of TLRs used as adjuvants in breast cancer vaccines are TLR9 ligand (CpG-oligonucleotides), TLR5 ligand (flagellin), TLR4 ligand (bacterial lipopolysaccharide (LPS)), and TLR3 ligand (Poly-ICLC).

TLRs have been found to be convincing immunostimulatory and are often co-delivered within the vaccine. TLR agonists were designed together within the peptide to overcome the poor immunogenicity of the peptide-based vaccine. To date, the incorporation of TLR agonists into a peptide has provided promising approaches for the development of an efficacious vaccine by targeting APC uptake and PRRs, thus allowing vaccines to achieve potent cell-mediated and humoral immunities [118]. As shown in many studies, the use of TLR-adjuvants helps in a way to increase the immunogenicity of a peptide [118,119,120]. In an in vitro study combining CpG oligonucleotides with cage protein, EP2 was able to increase MHC 1 display and induce CD8 T cell activation at a dose lower than required for DC maturation. The combination also increased MHC I display and CD8 T cell activation relative to unbound forms of the individual components. 

Adjuvants need to follow pre-selected requirements, including being non-toxic, capable of protecting peptides from rapid degradation, stimulating a good humoral/T-cell response, and not causing autoimmune or allergic reactions [120,121,122]. Examples of widely used adjuvants include Montanide ISA-51 (IFA), poly I: C, GpG ODN, TLR-dependent, AS15, Freund’s complete adjuvant (FCA), and GM-CSF. A combination of adjuvants may eventually be essential as it can modulate a potent immune response and intensify T helper cells as if one adjuvant were low. CpG-ODN induces the proliferation of B cells, activation of macrophages, and thus stimulation of the immune system, as in tetanus toxoid. CpGODN co-administration with antigens creates potent immunogens in vivo and in vitro for novel vaccine delivery [123].

Recent clinical trials on fifty-one patients reported by Melssen and team show LPS and poly-ICLC are safe and effective vaccine adjuvants even when combined with incomplete Freund’s adjuvant (IFA), thus conferring protection against melanoma [124]. In fact, the results demonstrated that a multi-peptide vaccine with TLR agonists enhanced T-cell responses against the pathogen, which is otherwise difficult to achieve, especially without an adjuvant. The multi-peptide-based vaccines can also plausibly be designed to include multiple epitopes from more than one antigen.

To date, four TLRs agonists have been approved by the FDA for cancer treatment which are monophosphoryl lipid A (MPLA), IMQ, and REQ [125]. Among them, Bacillus Calmette-Guerin (BCG) was the first FDA-licensed TLR-based adjuvant used in bladder cancer vaccines. BCG is a mixture of TLR2/TLR4 agonists derived from *Mycobacterium bovis* bacteria that was previously used against tuberculosis. The licensed adjuvants are considered few despite extensive research and technology, and surprisingly, there is no information about standard adjuvants used for peptide-based vaccines against breast cancer. Hence, there is a necessity to explore the standard combination of vaccine-adjuvants that is effective.

Conjugation of the peptide sequence with immunostimulatory molecules such as TLRs and encapsulation with cancer antigens would enhance killing mechanisms against the tumor and improve its immunological effectiveness by targeting APC-expressed PRRs and allowing vaccines to achieve effective and long-term immunity [126]. For instance, it also improves the stability and reproducibility of vaccines besides amplifying the onset of an immune response [127,128]. 

#### 5.2.2. Granulocyte Macrophage-Colony Stimulating Factor (GM-CSF)

GM-CSF is a type of cytokine derived from the activation of several types of cells, including T cells, B cells, macrophages, fibroblasts, monocytes, mast cells, and endothelial cells [129]. GM-CSF improves the role of APCs by activating, maturing, and conscribing dendritic cells (DCs), macrophages, eosinophils, and monocytes [130]. GM-CSF is a potent chemotactic factor that increases the expression of pro-inflammatory mediators such as IL-1 and TNF-, which in turn up-regulates GM-CSF itself [131]. Owing to this reason, GM-CSF has the potential to be explored as an immunostimulatory substance in various conditions such as autoimmunity, inflammation, and cancer [132,133,134,135]. In preclinical studies, the use of GM-CSF as an immunostimulant agent has been shown to evoke strong cell-mediated immune responses, thus suppressing tumor growth [136,137]. 

#### 5.2.3. Keyhole Limpet Hemocyanin (KLH)

Keyhole limpet hemocyanin (KLH) is a potent immunogenic protein carrier that is able to induce both T cell and B cell production in animals and humans [138,139]. It is derived from the hemolymph of the inedible sea mollusk, *Megathura crenulata*. KLH was first introduced to patients in 1967 to determine the immunologic responsiveness of individuals undergoing immunosuppressive therapy with amethopterin or azathioprine [140,141]. KLH has also been used clinically as a carrier and adjuvant for the vaccine. Riggs and colleagues have demonstrated that KLH itself is able to inhibit the growth of ZR75-1, MCF-7, and PANC-1 cancer cell lines in vitro by an average of more than 30%. KLH acts as an immune stimulant when used as a conjugate vaccine, especially for peptide- and carbohydrate-based vaccines [142]. A current study from Wimmers et al. [138] monitored B cell responses to KLH in Stage III melanoma patients. The study found that a massive >100-fold expansion of CD19+ B cells was observed in all patients analyzed. Based on many previous studies, it can be concluded that KLH is a promising immunostimulant agent.

## 6. Selection of Main TAA-Derived Peptide Antigens

Peptides applied in the breast cancer vaccines have described in the Table 1. The peptides include GP2 peptide, peptide I-6, P5 peptide, MUC1-specific peptide vaccine sequence APGSTAPPA and SAPDTRPAP, E75 peptide, p5 HER-2/neu derived peptide and long peptide (conjugating SU18 peptide with SU22 peptide using glycine linker). Table 2 summarized the selected breast cancer peptides in the clinical trials.

**Table 1:** Peptides applied in breast cancer vaccines.

Peptides	Mechanism of Action	Types of Study	Results	Ref.
GP2 Peptide	Stimulates helper T cells, cytotoxic T lymphocytes, and antibodies	In vivo study in xenograft mice using TUBO cells	GP2 peptide alone did not have a significant therapeutic and prophylactic effect in mice	[143]
Peptide I-6	Targets MAGE-1 on breast cancer, thus, inducing the antitumor effect from CTLs	In vitro study: MDA-MB-231 cells In vivo study: MCF-7 cells	I-6 induced cytotoxic activity against MDA-MB-231 cells by activating CD8þ T lymphocytes	[32]
P5 peptide (HER-2 derived peptide)	P5 peptide releases a high amount of IFN-γ and IL-10, therefore, inducing a potent CTL immune response	In vivo: induce TUBO cells in BALB/c mice	P5 peptide conjugated with maleimide-PEG2000-DSPE incorporation into liposomes stimulate immunogenicity and anti-tumour activities more potent than P5 peptide alone	[109]
MUC1-specific peptide vaccine sequence APGSTAPPA and SAPDTRPAP	The peptide induces IFN-γ-producing T cells	In vitro MTag cell lines In vivo: Mammary gland tumors from PyV MT mice PyV MT mice	Immunosuppression within the tumor microenvironment hinders the immune response to anti-cancer vaccines	[144]
E75 Peptide, also known as p369 peptide	Ability to bind specific CD8^+^ TL clones that could lyse HER2-positive tumor cells	In vitro breast cancer cell lines; MCF-7, MDA-MB-231 In vivo mice model Clinical trial	Two Phase-II clinical trials on patients resulted in remission after breast cancer but were considered at high risk of recurrence.	[52,145,146,147]
p5 HER-2/neu derived peptide	Induce a high level of CD8+ CTL, which is capable of killing tumor cells via recognizing the TAAs epitopes presented on the surface of cancer cells in association with MHC I molecules.	In vitro: TUBO cell In vivo: Female BALB/c mice were subcutaneously administered at the right flank	Free p5 peptide showed weak antitumor and CTLs response activities compared to Liposome–DOPE–p5 + CpG-ODN formulation	[65]
Long peptide (conjugating SU18 peptide with SU22 peptide using glycine linker)	The long peptides (containing T helper and killer epitope) targeted the overexpression of Survivin antigens in breast cancer cells.	Clinical trial (Phase 1). The vaccine was given every two weeks for 4 times.	A customized peptide with multiple epitopes and containing a long sequence of amino acids provide superior and innovative cancer vaccine designs, which are capable of inducing both Th1 and Th2 immune responses in cancer patients.	[148]

**Table 2:** Summary selected breast cancer peptides in clinical trials.

Agent	Phase	Adjuvant	Enrolment	Regime of Treatment	Ref.
NeuVax™ (Nelipepimut-S or E75)	III	Leukine^®^ [sargramostim, GM-CSF]	758 patients	Once a month, for six consecutive months, and then booster for every six months total of 36 months	[52]
HER-2/*neu* ECD & ICD Peptides	I	Granulocyte-macrophage colony-stimulating factor (GM-CSF)	8 patients	Once a month for 2–6 months, intradermally	[149]
Folate Receptor Alpha (FRα) peptide vaccine	II	GM-CSF	80 patients	Single ID administration—monthly vaccinations repeated six times, followed by boosters every six months until recurrence.	[150,151,152]
MUC-1 peptide vaccine	I	poly-ICLC	29 patients	Subcutaneous (SC) injection in weeks 0, 2, and 10.	[153]
AE37 Peptide Vaccine	II	GMCSF	600 patients	Intradermally (ID) injection every 3–4 weeks for a total of up to 6 inoculations followed up every 3 months for the first 2 years.	[154]
E39 and J65 peptide vaccine	I	GMCSF	39 patients	Receive six monthly injections of peptide + GM-CSF booster inoculation within 1–2 weeks of their 6-month period	[155]
hTERT/Survivin Multi-Peptide Vaccine	1	-	11 patients	Receive subcutaneous injection every two weeks four times, then monthly up to 28 vaccinations, then every six months	[156]
WT1 peptide-based	I	Montanide ISA51	2 patients	Receive WT1 peptide intradermally three times at 2-week intervals	[157,158]
Ii-Key hybrid HER-2/*neu* peptide (AE37) vaccine	I	GM-CSF	15 patients	Receive vaccine via intradermal injection for six months	[159]

## 7. Nanoparticles as Peptide Vaccine Delivery Platform

Conjugation at the peptide terminals or encapsulation with liposomes, nanoparticles, immune-stimulating complexes (ISCOMs), and hydrogel has been extensively explored as an ideal delivery platform that can protect the peptide from degradation without jeopardizing its efficacy and safety. This effort could be synchronized with adjuvants to develop successful vaccines. Moreover, these manipulations are capable of increasing the APCs uptake, resulting in improved binding to MHC or T cell receptor sensitivity (TCR) and improving the secretion of interleukin-2 (IL-2) molecules and other mediators for CD8+ and CD4+ activation. As a consequence, the use of conjugation and encapsulation of nanoparticles tends to enhance the biostability and delivery system at the target site and make the vaccine more potent. 

Liposomes are phospholipids or lipid bilayers that form closed membrane vesicles. Many researchers have proposed the use of liposomes in peptide-based vaccine development due to their good encapsulation efficiency, biodegradability, biocompatibility, and non-toxicity. Liposomes can be integrated or intercalated with lipophilic and hydrophilic peptides [160]. Moreover, liposomes have a long circulation in the body, and this provides an advantage for cellular uptake with APCs. 

Integration of peptides onto the surface of the liposomes has become a famous approach in vaccine delivery due to its effectiveness. With regard to breast cancer vaccines, Razazan and colleagues developed a vaccine from HER2/neu-derived peptides using liposomes as a carrier delivery [143]. This lipopeptide vaccine induces a high CTL immune response and prolonged survivability in the BALB/c mouse model.

In another study, the use of cationic liposomes anchored with proteins conjugated with CpGODN elicited CD4+ T-cell responses. The positive charge benevolence formation of the depot action at the injection site is followed by a sustained release to the draining lymph nodes [123,161].

Nanoparticles (NPs) serve as a delivery platform in many vaccine formulations. The particle size, surface charge, and antigen loading mode of NPs greatly influence the adjuvanticity and immunoreactivity effects of potent and smooth vaccine delivery systems. Examples of famous nanomaterials that have continuously gained attention in the preparation of peptide-based vaccines are PLGA and chitosan. The strategy of using PLGA or chitosan as a vaccine delivery system has gained popularity among researchers. Both are types of polymers that are well-known as being non-harmful, non-toxic, biodegradable, and biocompatible. These nano-polymers, when incorporated with a peptide vaccine, can protect the peptide from degradation by proteolytic enzymes, thus providing more effective uptake and delivery to the lymphatic system [162]. Moreover, it could enhance and regulate immune responses.

Encapsulation of peptide antigens in polymeric particulates provides greater access to barrier compartments such as endothelial cell junctions, including the blood–brain barrier, when designed in a nanosize range of 120 nm; thus, this can be exploited for vaccine formulations, while sizes outside of this range are more likely to be blocked and cleared from the circulation [163,164]. PLGA, composed of two monomers, polylactic acid (LA) and polyglycolic acid, are the most ardent and ideal transport options for biomolecules as they are moist, biodegradable, biocompatible polymers and sturdy materials for medicinal and cosmetic applications [165]. They are metabolized to H_2_O and CO_2_ as end products before they are eliminated from the body.

To date, it is the most favored polymer to be used for drug delivery and is capable of protecting peptides from enzymatic degradation due to its ability to avoid the endosomal pouch. Peptide encapsulation into PLGA nanoparticles would provide a solid shield and efficiently improve drug release. Kroll et al. [166] demonstrated the ability of membrane-coated nanoparticle cancer cells to cause multi-antigenic antitumor immunity and extend the lives of mice with melanoma.

Gu et al. [167] reported that peptides coated with PLGA could protect peptide antigens from degradation by proteases and lysosomes and co-deliver the peptide antigen uptake by the APCs. Peptide antigen encapsulated in PLGA displayed slow antigen release. This process elicits immunological responses due to adequate antigen uptake by APCs, which express the fragments on their surface and then cross-present them through the MHC-binding complex pathway. Binding with MHC class 1 mostly activates cell-mediated immunity, while antigen binding with MHC class II optimally triggers an antibody response [168]. PLGA has been chosen as a compound delivery vehicle due to its wide safety profile and has been licensed by the FDA for medical applications [169,170]. Co-delivery of vaccine formulations in PLGA has shown promising results. For instance, Ma and co-researchers have developed a DC-based vaccine loaded with PLGA-NPs encapsulating the mSTEAP peptide against adenocarcinoma in C57BL/6 mice. The result was that PLGA NPs mediated good platform delivery and elicited strong immune responses in vivo [171].

Chen and colleagues have developed a combination of PLGA-ICG-R837, which is photothermal therapy with checkpoint blockade, adjuvant, and nanoparticles, against 4T1 mouse mammary carcinoma in BALB/c mice [172]. Synergistic anticancer effects were triggered by the combination of PLGA-ICG-R837 and checkpoint blockage, which induce potent immune responses from CTLs, thus suppressing tumor growth. This result indicated the benefit of using that combination, which may not just suppress cancer growth but also confer lifelong protection to prevent tumor relapse. Interestingly, the NPs and adjuvants used are FDA-approved ones, which have a good prospect for clinical translation.

Chu et al. [173] reported the potential of using chitosan NPs as a vaccine carrier for peptide-based vaccines due to their adjuvant effects as well as their stability properties in an acidic environment [174,175], which are suitable in breast cancer environments. Jadidi-Niaragh demonstrated that a dendritic cell vaccine incorporated with chitosan-lactate nanoparticles (ChLa NPs) inhibits metastasis and suppresses tumor growth, thus improving mice’s survival [176]. The incorporation of chitosan NPs into vaccine formulation was proven to promote DC activation and trigger robust cell-mediated immunity, which can seemingly be applied to cancer therapy [177]. With this evidence, such approaches can be employed and exploited for the purpose of fighting breast cancer.

As a consequence, the use of peptide nanoparticles camouflaged with cancer cell membranes has become a new and interesting strategy in vaccinology. Fang et al. [178] developed a cancer vaccine by utilizing biodegradable polymeric nanoparticles cloaked with the MDA-MB-435 cell membrane, and a monophosphoryl lipid A-TLR-4 activator was used as an adjuvant. The cancer cell membrane-cloaked NPs vaccine demonstrated high dendritic cell maturation, which resulted in higher secretion of IFN. It is believed that MDA-MB-435 overexpressed galectin-3 and CEA, thus triggering this vaccine’s antitumor response via the homologous binding mechanism. The idea was further explored by Chen et al. [172], which encapsulated PLGA-NPs with MDA-MB-231 and Hela cell line fragments. Doxorubicin and PD-L1 siRNA checkpoint inhibitors were loaded. The anticancer drug is capable of detecting cancer with an accurate and sensitive CTL response. As is currently the case, the restrictions on the use of most anticancer drugs do not provide long-lasting protection and have limited circulatory duration. Instead of using anticancer drugs, perhaps the methods can be shifted to peptide-based materials, which also have anticancer activity.

## 8. Future Direction: Rational Vaccine Design

### 8.1. Biomimetic Nano-Peptide Vaccine

The comprehensive advancement of the nanoparticle delivery system has driven significant improvements in cancer therapy. Biomimetic nanotechnology has recently attracted attention with its notion of wrapping polymeric nanoparticles (NPs) with cell membranes; they were extracted from breast cancer cells. This has resulted in multi-immune responses to different tumor antigens attributable to receptor-ligand interactions in surface cells [179,180], thereby enhancing biological adhesion and immune clearance [181]. This follows the weak efficacy of a single antigenic determinant vaccine, which lacks immune defenses, particularly when confronted with mutated and heterogeneous cancer cells. 

**Figure 3:**
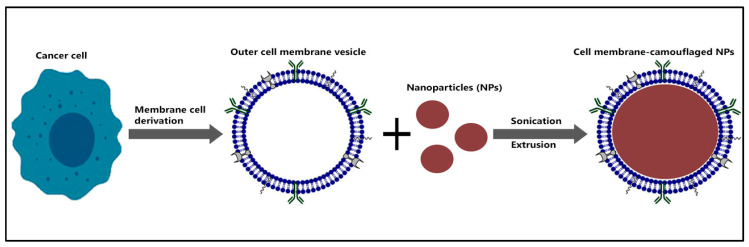
Schematic illustration of the preparation of cancer cell membrane-camouflaged nanoparticles. The intracellular contents of the cell can be extracted to form a ‘ghost cancer cell’ by applying hypotonic treatment. The NPs can be fused through sonication followed by extrusion.

The cell membrane is coated to preserve the biological function of the vaccine in inducing a robust and precise immune response. ‘Ghost cancer cell membrane’ is a term that has been used to extract intracellular cell material and preserve the cell membrane. The contents can be critically extracted by incubation in a hypotonic solution accompanied by sonication and by co-extrusion of the cell membrane and nanoparticle with the extruder. The illustration is shown in Figure 3.

This biomimetic concept can be adopted and further improved for peptide-based vaccine technology. Research by Jin et al. [182] showed the applicability of this biomimetic development to cancer immunotherapy-coated primary human glioblastoma cell membrane fractions (U87 and U87-CXCR4 cell lines) in PLGA NPs. The results showed that CD4+ and CD8+ T lymphocytes in the lymph nodes and spleens of the Balb/cc mouse model were activated, and the metastatic burden was significantly reduced. Future insights on the use of biomimetic technologies for breast cancer vaccinations are highly exciting and yet emerging.

### 8.2. Combining Immune Checkpoint Blockade Agents with Peptide-Based Vaccine

Similar to peptide-based cancer vaccines, to date, none of the currently available vaccinations can provide complete protection. With few side effects, it may be useful in cases of early cancer detection for preventing relapse or enhancing survival. Therefore, combinatorial treatments, on the other hand, may be able to successfully treat even advanced cancers while also overcoming immune escape mechanisms and tumor-mediated immunosuppression issues [183,184]. For example, by combining with immune checkpoint blockade therapies such as programmed cell death-1 (PD-1) and CTLA-4 targeted antibodies. 

The vital importance of an efficient immune response for controlling cancerous cells was discussed when scientists found out that breast cancer is also a type of immunogenic disease [26,185]. Additionally, there is strong evidence indicating a link between a beneficial outcome in different malignancies and tumor-infiltrating lymphocytes (TILs) in tumor tissue [186,187]. The PD-1 receptor interacts with its ligand (either PD-L1 or PD-L2) on cancer cells, resulting in immune checkpoint pathway activation. When PD-1 is overexpressed on T cells, B cells, and NK cells, those immune cells are suppressed and deactivated, hijacked by tumors [188,189]. Anti-PD-1 agents have shown promising results in the metastatic environment, while combination strategies tend to enhance more responses [188]. In animal models, a combination of a peptide-based vaccine with CTLA-4, specific to T-cell inhibitory receptors, has shown remarkable activation of tumor-specific T cells against cancerous cells [190].

Several studies pertaining to combinational therapy with cancer vaccines and PD-1 were reported. The explanation for this is that some cancer patients do not respond to anti-PD-1 agents due to a lack of TILs and cancer vaccines that induce effector T-cell infiltration into the tumors. Thus, this provides a convincing concept that anti-PD-1/PD-L1 agents may act synergistically to induce a stronger immune response against the tumor [191]. 

## 9. Conclusions

Overall, the development of peptide-derived cancer antigen vaccines is encouraging because of their advantages in identifying target antigens and their ability to select appropriate adjuvants to combine to enhance immunogenicity. It opens a window for future breast cancer therapy. Further focus should be given to their experimental translation and their clinical implementation. The weak immunogenicity issue of the peptide can be improved with coadministration with an immunostimulatory agent such as KLH, GM-CSF, CpG-ODN, or poly:IC because the most important value of using peptides from an experimental point of view is their effectiveness with a wide safety margin. 

Additionally, the peptide antigen itself can be chemically modified. For example, the lipopeptides derived from microbes can self-assemble into nanoparticles due to their amphiphilic properties. Changes in the microenvironment of breast cancer (poor tumor vasculature, hypoxia, acidic area) plus immunosuppression of the patient require improvement in peptide-based therapy that perhaps has antimicrobial properties, preventing secondary bacterial infections.

Intercalating the modification with multiple antigens and immunostimulatory substances would offer a potent immune response from the innate and adaptive immune systems. Moreover, multiple antigenic and chimeric peptide approaches offer the opportunity for antigenic peptides from various TAAs and CTL peptide epitopes to be covalently combined in a single system. In order to render their biological functions, the designated multiple peptides, antibodies, and nanoparticles can be further incorporated into the functionalized eukaryotic cell membrane. Owing to the cancer cell’s antigenic diversity, membrane-coated nanoparticles are expected to be promising and can be used specifically to target breast cancer. Numerous cancer-associated moieties—proteins, ligands, and receptors—are found in the vaccine formulation from the use of peptides extracted from breast cancer. Among others, there must be a few or an entire group that is responsible for effective targeting, which can kill two birds with one stone. The concept of using a peptide-based vaccine for breast cancer therapy is fashionable and possible to accomplish. The more complex and comprehensive the system, the less chance there is for the cancer cells to escape. 

## Data Availability

Data is contained within the article.

## References

[1] World Health Organization Breast Cancer: Prevention and Control. https://www.who.int/cancer/prevention/diagnosis-screening/breast-cancer/en/.

[2] Reddington R., Galer M., Hagedorn A., Liu P., Barrack S., Husain E., Sharma R., Speirs V., Masannat Y. (2020). Incidence of Male Breast Cancer in Scotland over a Twenty-Five-Year Period (1992–2017). Eur. J. Surg. Oncol..

[3] Giordano S.H. (2018). Breast Cancer in Men. N. Engl. J. Med..

[4] Gargiulo P., Pensabene M., Milano M., Arpino G., Giuliano M., Forestieri V., Condello C., Lauria R., De Placido S. (2016). Long-Term Survival and BRCA Status in Male Breast Cancer: A Retrospective Single-Center Analysis. BMC Cancer.

[5] Gaddam S., Heller S.L., Babb J.S., Gao Y. (2021). Male Breast Cancer Risk Assessment and Screening Recommendations in High-Risk Men Who Undergo Genetic Counseling and Multigene Panel Testing. Clin. Breast Cancer.

[6] Siegel R.L., Miller K.D., Jemal A. (2015). Cancer Statistics, 2015. CA Cancer J. Clin..

[7] Shah R., Rosso K., David Nathanson S. (2014). Pathogenesis, Prevention, Diagnosis and Treatment of Breast Cancer. World J. Clin. Oncol..

[8] Ly D., Forman D., Ferlay J., Brinton L.A., Cook M.B. (2013). An International Comparison of Male and Female Breast Cancer Incidence Rates. Int. J. Cancer.

[9] Chakraborty S., Rahman T. (2012). The Difficulties in Cancer Treatment. Ecancermedicalscience.

[10] Ekici S., Jawzal H. (2020). Breast Cancer Diagnosis Using Thermography and Convolutional Neural Networks. Med. Hypotheses.

[11] Sánchez-Bermúdez A.I., Sarabia-Meseguer M.D., García-Aliaga A., Marín-Vera M., Macías-Cerrolaza J.A., Henaréjos P.S., Guardiola-Castillo V., la Peña F.A.-D., Alonso-Romero J.L., Noguera-Velasco J.A. (2018). Mutational Analysis of RAD51C and RAD51D Genes in Hereditary Breast and Ovarian Cancer Families from Murcia (Southeastern Spain). Eur. J. Med. Genet..

[12] Karami F., Mehdipour P. (2013). A Comprehensive Focus on Global Spectrum of BRCA1 and BRCA2 Mutations in Breast Cancer. Biomed. Res. Int..

[13] Ayala de la Peña F., Andrés R., Garcia-Sáenz J.A., Manso L., Margelí M., Dalmau E., Pernas S., Prat A., Servitja S., Ciruelos E. (2019). SEOM Clinical Guidelines in Early Stage Breast Cancer (2018). Clin. Transl. Oncol..

[14] Suter R., Marcum J.A. (2007). The Molecular Genetics of Breast Cancer and Targeted Therapy. Biologics.

[15] Townsend D.M., Tew K.D. (2003). The Role of Glutathione-S-Transferase in Anti-Cancer Drug Resistance. Oncogene.

[16] Aziz M.H., Ahmad A. (2020). Epigenetic Basis of Cancer Drug Resistance. Cancer Drug Resist..

[17] Qin S., Xu L., Yi M., Yu S., Wu K., Luo S. (2019). Novel Immune Checkpoint Targets: Moving beyond PD-1 and CTLA-4. Mol. Cancer.

[18] Ji X., Lu Y., Tian H., Meng X., Wei M., Cho W.C. (2019). Chemoresistance Mechanisms of Breast Cancer and Their Countermeasures. Biomed. Pharmacother..

[19] Crespo I., Götz L., Liechti R., Coukos G., Doucey M.A., Xenarios I. (2016). Identifying Biological Mechanisms for Favorable Cancer Prognosis Using Non-Hypothesis-Driven Iterative Survival Analysis. NPJ Syst. Biol. Appl..

[20] Anfray C., Ummarino A., Andón F.T., Allavena P. (2020). Current Strategies to Target Tumor-Associated-Macrophages to Improve Anti-Tumor Immune Responses. Cells.

[21] Davis R.J., Van Waes C., Allen C.T. (2016). Overcoming Barriers to Effective Immunotherapy: MDSCs, TAMs, and Tregs as Mediators of the Immunosuppressive Microenvironment in Head and Neck Cancer. Oral Oncol..

[22] Lin Y., Xu J., Lan H. (2019). Tumor-Associated Macrophages in Tumor Metastasis: Biological Roles and Clinical Therapeutic Applications. J. Hematol. Oncol..

[23] Schmitt N., Bustamante J., Bourdery L., Bentebibel S.E., Boisson-Dupuis S., Hamlin F., Tran M.V., Blankenship D., Pascual V., Savino D.A. (2013). IL-12 Receptor Β1 Deficiency Alters in Vivo T Follicular Helper Cell Response in Humans. Blood.

[24] Lindau D., Gielen P., Kroesen M., Wesseling P., Adema G.J. (2013). The Immunosuppressive Tumour Network: Myeloid-Derived Suppressor Cells, Regulatory T Cells and Natural Killer T Cells. Immunology.

[25] Li S., Yao M., Niu C., Liu D., Tang Z., Gu C., Zhao H., Ke J., Wu S., Wang X. (2019). Inhibition of MCF-7 Breast Cancer Cell Proliferation by a Synthetic Peptide Derived from the C-Terminal Sequence of Orai Channel. Biochem. Biophys. Res. Commun..

[26] Gatti-Mays M.E., Balko J.M., Gameiro S.R., Bear H.D., Prabhakaran S., Fukui J., Disis M.L., Nanda R., Gulley J.L., Kalinsky K. (2019). If We Build It They Will Come: Targeting the Immune Response to Breast Cancer. NPJ Breast Cancer.

[27] Gun S.Y., Lee S.W.L., Sieow J.L., Wong S.C. (2019). Targeting Immune Cells for Cancer Therapy. Redox Biol..

[28] Zhang Y., Zhang Z. (2020). The History and Advances in Cancer Immunotherapy: Understanding the Characteristics of Tumor-Infiltrating Immune Cells and Their Therapeutic Implications. Cell Mol. Immunol..

[29] Criscitiello C., Viale G., Curigliano G. (2019). Peptide Vaccines in Early Breast Cancer. Breast.

[30] Yamaguchi Y., Yamaue H., Okusaka T., Okuno K., Suzuki H., Fujioka T., Otsu A., Ohashi Y., Shimazawa R., Nishio K. (2014). Guidance for Peptide Vaccines for the Treatment of Cancer. Cancer Sci..

[31] Waks A.G., Winer E.P. (2019). Breast Cancer Treatment: A Review. JAMA J. Am. Med. Assoc..

[32] Shi W., Qiu Q., Tong Z., Guo W., Zou F., Feng Z., Wang Y., Huang W., Qian H. (2020). Synthetic Tumor-Specific Antigenic Peptides with a Strong Affinity to HLA-A2 Elicit Anti-Breast Cancer Immune Response through Activating CD8+ T Cells. Eur. J. Med. Chem..

[33] Nguyen T.L., Choi Y., Kim J. (2019). Mesoporous Silica as a Versatile Platform for Cancer Immunotherapy. Adv. Mater..

[34] De Temmerman M.L., Rejman J., Demeester J., Irvine D.J., Gander B., De Smedt S.C. (2011). Particulate Vaccines: On the Quest for Optimal Delivery and Immune Response. Drug Discov. Today.

[35] Nejad A.E., Najafgholian S., Rostami A., Sistani A., Shojaeifar S., Esparvarinha M., Nedaeinia R., Javanmard S.H., Taherian M., Ahmadlou M. (2021). The role of hypoxia in the tumor microenvironment and development of cancer stem cell: A novel approach to developing treatment. Cancer Cell Intl..

[36] Solinas C., Aiello M., Migliori E., Willard-Gallo K., Emens L.A. (2020). Breast cancer vaccines: Heeding the lessons of the past to guide a path forward. Cancer Treat. Rev..

[37] de Paula Peres L., da Luz F.A.C., dos Anjos Pultz B., Brígido P.C., de Araújo R.A., Goulart L.R., Silva M.J.B. (2015). Peptide Vaccines in Breast Cancer: The Immunological Basis for Clinical Response. Biotechnol. Adv..

[38] Criscitiello C. (2012). Tumor-Associated Antigens in Breast Cancer. Breast Care.

[39] Thundimadathil J. (2012). Cancer Treatment Using Peptides: Current Therapies and Future Prospects. J. Amino Acids.

[40] Blum J.S., Wearsch P.A., Cresswell P. (2013). Pathways of Antigen Processing. Annu. Rev. Immunol..

[41] Zhang L., Huang Y., Lindstrom A.R., Lin T.Y., Lam K.S., Li Y. (2019). Peptide-Based Materials for Cancer Immunotherapy. Theranostics.

[42] Santoni D. (2018). Viral Peptides-MHC Interaction: Binding Probability and Distance from Human Peptides. J. Immunol. Methods.

[43] Jones N.D., Van Maurik A., Hara M., Spriewald B.M., Witzke O., Morris P.J., Wood K.J. (2000). CD40-CD40 Ligand-Independent Activation of CD8+ T Cells Can Trigger Allograft Rejection. J. Immunol..

[44] Tay N.Q., Lee D.C.P., Chua Y.L., Prabhu N., Gascoigne N.R.J., Kemeny D.M. (2017). CD40L Expression Allows CD8+ T Cells to Promote Their Own Expansion and Differentiation through Dendritic Cells. Front. Immunol..

[45] Wong K.L., Tang L.F.M., Lew F.C., Wong H.S.K., Chua Y.L., MacAry P.A., Kemeny D.M. (2009). CD44high Memory CD8 T Cells Synergize with CpG DNA to Activate Dendritic Cell IL-12p70 Production. J. Immunol..

[46] Ikeda H., Shiku H. (2012). Immunotherapy of Solid Tumor: Perspectives on Vaccine and Cell Therapy. Nihon Rinsho.

[47] Costa R.L.B., Czerniecki B.J. (2020). Clinical Development of Immunotherapies for HER2+ Breast Cancer: A Review of HER2-Directed Monoclonal Antibodies and Beyond. NPJ Breast Cancer.

[48] Lyu H., Huang J., He Z., Liu B. (2018). Epigenetic Mechanism of Survivin Dysregulation in Human Cancer. Sci. China Life Sci..

[49] Genitsch V., Zlobec I., Thalmann G.N., Fleischmann A. (2016). MUC1 Is Upregulated in Advanced Prostate Cancer and Is an Independent Prognostic Factor. Prostate Cancer Prostatic. Dis..

[50] Malonis R.J., Lai J.R., Vergnolle O. (2020). Peptide-Based Vaccines: Current Progress and Future Challenges. Chem. Rev..

[51] Kim I., Sanchez K., McArthur H.L., Page D. (2019). Immunotherapy in Triple-Negative Breast Cancer: Present and Future. Curr. Breast Cancer Rep..

[52] Mittendorf E.A., Lu B., Melisko M., Hiller J.P., Bondarenko I., Brunt A.M., Sergii G., Petrakova K., Peoples G.E. (2019). Efficacy and Safety Analysis of Nelipepimut-S Vaccine to Prevent Breast Cancer Recurrence: A Randomized, Multicenter, Phase III Clinical Trial. Clin. Cancer Res..

[53] Jackson Doreen O., Francois T.A., Travis C.G., Vreeland Timothy J., Peace Kaitlin M., Hale Diane F., Litton Jennifer K., Murray James L., Perez Sonia A., Michael P. (2018). Effects of HLA Status and HER2 Status on Outcomes in Breast Cancer Patients at Risk for Recurrence—Implications for Vaccine Trial Design. Clin. Immunol..

[54] Patil R., Clifton G.T., Holmes J.P., Amin A., Carmichael M.G., Gates J.D., Benavides L.H., Hueman M.T., Ponniah S., Peoples G.E. (2010). Clinical and Immunologic Responses of HLA-A3+ Breast Cancer Patients Vaccinated with the HER2/Neu-Derived Peptide Vaccine, E75, in a Phase I/II Clinical Trial. J. Am. Coll. Surg..

[55] Gourley C., Thornton C., Massie C., Prescott R.J., Turner M., Leonard R.C.F., Kilpatrick D.C. (2003). Is There a Relationship between HLA Type and Prognostic Factors in Breast Cancer?. Anticancer Res..

[56] Mahjoubin-Tehran M., Rezaei S., Jalili A., Aghaee-Bakhtiari S.H., Orafai H.M., Jamialahmadi T., Sahebkar A. (2020). Peptide Decoys: A New Technology Offering Therapeutic Opportunities for Breast Cancer. Drug Discov. Today.

[57] Farzad N., Barati N., Momtazi-Borojeni A.A., Yazdani M., Arab A., Razazan A., Shariat S., Mansourian M., Abbasi A., Saberi Z. (2019). P435 HER2/Neu-Derived Peptide Conjugated to Liposomes Containing DOPE as an Effective Prophylactic Vaccine Formulation for Breast Cancer. Artif. Cells Nanomed. Biotechnol..

[58] Furrer D., Sanschagrin F., Jacob S., Diorio C. (2015). Advantages and Disadvantages of Technologies for HER2 Testing in Breast Cancer Specimens: Table 1. Am. J. Clin. Pathol..

[59] Slamon D.J., Clark G.M., Wong S.G., Levin W.J., Ullrich A., McGuire W.L. (1987). Human Breast Cancer: Correlation of Relapse and Survival with Amplification of the HER-2/Neu Oncogene. Science.

[60] Ayoub N.M., Al-Shami K.M., Yaghan R.J. (2019). Immunotherapy for HER2-Positive Breast Cancer: Recent Advances and Combination Therapeutic Approaches. Breast Cancer Targets Ther..

[61] Krasniqi E., Barchiesi G., Pizzuti L., Mazzotta M., Venuti A., Maugeri-Saccà M., Sanguineti G., Massimiani G., Sergi D., Carpano S. (2019). Immunotherapy in HER2-Positive Breast Cancer: State of the Art and Future Perspectives. J. Hematol. Oncol..

[62] Katzorke N., Rack B.K., Haeberle L., Neugebauer J.K., Melcher C.A., Hagenbeck C., Forstbauer H., Ulmer H.U., Soeling U., Kreienberg R. (2013). Prognostic Value of HER2 on Breast Cancer Survival. J. Clin. Oncol..

[63] Clynes R.A., Towers T.L., Presta L.G., Ravetch J.V. (2000). Inhibitory Fc Receptors Modulate in Vivo Cytoxicity against Tumor Targets. Nat. Med..

[64] Wang J., Xu B. (2019). Targeted Therapeutic Options and Future Perspectives for Her2-Positive Breast Cancer. Signal Transduct. Target. Ther..

[65] Mansourian M., Badiee A., Jalali S.A., Shariat S., Yazdani M., Amin M., Jaafari M.R. (2014). Effective Induction of Anti-Tumor Immunity Using P5 HER-2/Neu Derived Peptide Encapsulated in Fusogenic DOTAP Cationic Liposomes Co-Administrated with CpG-ODN. Immunol. Lett..

[66] Costa R.L.B., Soliman H., Czerniecki B.J. (2017). The Clinical Development of Vaccines for HER2+ Breast Cancer: Current Landscape and Future Perspectives. Cancer Treat. Rev..

[67] Richman C.M., DeNardo S.J. (2001). Systemic Radiotherapy in Metastatic Breast Cancer Using 90Y-Linked Monoclonal MUC-1 Antibodies. Crit. Rev. Oncol. Hematol..

[68] Nath S., Mukherjee P. (2014). MUC1: A Multifaceted Oncoprotein with a Key Role in Cancer Progression. Trends Mol. Med..

[69] Kovjazin R., Horn G., Smorodinsky N.I., Shapira M.Y., Carmon L. (2014). Cell Surface-Associated Anti-MUC1-Derived Signal Peptide Antibodies: Implications for Cancer Diagnostics and Therapy. PLoS ONE.

[70] Lakshminarayanan V., Thompson P., Wolfert M.A., Buskas T., Bradley J.M., Pathangey L.B., Madsen C.S., Cohen P.A., Gendler S.J., Boons G.J. (2012). Immune Recognition of Tumor-Associated Mucin MUC1 Is Achieved by a Fully Synthetic Aberrantly Glycosylated MUC1 Tripartite Vaccine. Proc. Natl. Acad. Sci. USA.

[71] Xing P.X., Michael M., Apostolopoulos V., Prenzoska J., Marshall C., Bishop J., McKenzie I.F.C. (1995). Phase I Study of Synthetic MUC1 Peptides in Breast Cancer. Int. J. Oncol..

[72] Apostolopoulos V., Pietersz G.A., Tsibanis A., Tsikkinis A., Drakaki H., Loveland B.E., Piddlesden S.J., Plebanski M., Pouniotis D.S., Alexis M.N. (2006). Pilot Phase III Immunotherapy Study in Early-Stage Breast Cancer Patients Using Oxidized Mannan-MUC1 [ISRCTN71711835]. Breast Cancer Res..

[73] Jeong S., Park M.J., Song W., Kim H.S. (2020). Current Immunoassay Methods and Their Applications to Clinically Used Biomarkers of Breast Cancer. Clin. Biochem..

[74] Brantley-Sieders D.M., Zhuang G., Hicks D., Wei B.F., Hwang Y., Cates J.M.M., Coffman K., Jackson D., Bruckheimer E., Muraoka-Cook R.S. (2008). The Receptor Tyrosine Kinase EphA2 Promotes Mammary Adenocarcinoma Tumorigenesis and Metastatic Progression in Mice by Amplifying ErbB2 Signaling. J. Clin. Investig..

[75] Gökmen-Polar Y., Toroni R.A., Hocevar B.A., Badve S., Zhao Q., Shen C., Bruckheimer E., Kinch M.S., Miller K.D. (2011). Dual Targeting of EphA2 and ER Restores Tamoxifen Sensitivity in ER/EphA2-Positive Breast Cancer. Breast Cancer Res. Treat..

[76] Scarberry K.E., Dickerson E.B., McDonald J.F., Zhang Z.J. (2008). Magnetic Nanoparticle-Peptide Conjugates for in Vitro and in Vivo Targeting and Extraction of Cancer Cells. J. Am. Chem. Soc..

[77] Salem A.F., Wang S., Billet S., Chen J.F., Udompholkul P., Gambini L., Baggio C., Tseng H.R., Posadas E.M., Bhowmick N.A. (2018). Reduction of Circulating Cancer Cells and Metastases in Breast-Cancer Models by a Potent EphA2-Agonistic Peptide-Drug Conjugate. J. Med. Chem..

[78] Guo Z., He B., Yuan L., Dai W., Zhang H., Wang X., Wang J., Zhang X., Zhang Q. (2015). Dual Targeting for Metastatic Breast Cancer and Tumor Neovasculature by EphA2-Mediated Nanocarriers. Int. J. Pharm..

[79] Jha K., Shukla M., Pandey M. (2012). Survivin Expression and Targeting in Breast Cancer. Surg. Oncol..

[80] Altieri D.C. (2008). Survivin, Cancer Networks and Pathway-Directed Drug Discovery. Nat. Rev. Cancer..

[81] Ryan B.M., Konecny G.E., Kahlert S., Wang H.J., Untch M., Meng G., Pegram M.D., Podratz K.C., Crown J., Slamon D.J. (2006). Survivin Expression in Breast Cancer Predicts Clinical Outcome and Is Associated with HER2, VEGF, Urokinase Plasminogen Activator and PAI-1. Ann. Oncol..

[82] Tanaka K., Kanazawa T., Horiuchi S., Ando T., Sugawara K., Takashima Y., Seta Y., Okada H. (2013). Cytoplasm-responsive nanocarriers conjugated with a functional cell-penetrating peptide for systemic siRNA delivery. Intl. J. Pharm..

[83] Rodel F., Sprenger T., Kaina B., Liersch T., Rodel C., Fulda S., Hehlgans S. (2012). Survivin as a Prognostic/Predictive Marker and Molecular Target in Cancer Therapy. Curr. Med. Chem..

[84] Garg H., Suri P., Gupta J.C., Talwar G.P., Dubey S. (2016). Survivin: A Unique Target for Tumor Therapy. Cancer Cell Int..

[85] Tsuda N., Murayama K., Ishida H., Matsunaga K., Komiya S., Itoh K., Yamada A. (2001). Expression of a Newly Defined Tumor-Rejection Antigen SART3 in Musculoskeletal Tumors and Induction of HLA Class I-Restricted Cytotoxic T Lymphocytes by SART3-Derived Peptides. J. Orthop. Res..

[86] Miyagi Y., Sasatomi T., Mine T., Isomoto H., Shirouzu K., Yamana H., Imai N., Yamada A., Katagiri K., Muto A. (2001). Induction of Cellular Immune Responses to Tumor Cells and Peptides in Colorectal Cancer Patients by Vaccination with SART3 Peptides. Clin. Cancer Res..

[87] Sherman E.J., Mitchell D.C., Garner A.L. (2019). The RNA-Binding Protein SART3 Promotes MiR-34a Biogenesis and G1 Cell Cycle Arrest in Lung Cancer Cells. J. Biol. Chem..

[88] Timani K.A., Gyorffy B., Liu Y., Mohammad K.S., He J.J. (2018). Tip110/SART3 Regulates IL-8 Expression and Predicts the Clinical Outcomes in Melanoma. Mol. Cancer.

[89] Lee J.H., Lee S.W. (2017). The Roles of Carcinoembryonic Antigen in Liver Metastasis and Therapeutic Approaches. Gastroenterol. Res. Pract..

[90] Turriziani M., Fantini M., Benvenuto M., Izzi V., Masuelli L., Sacchetti P., Modesti A., Bei R. (2012). Carcinoembryonic Antigen (CEA)-Based Cancer Vaccines: Recent Patents and Antitumor Effects from Experimental Models to Clinical Trials. Recent. Pat. Anticancer Drug Discov..

[91] Ojima T., Iwahashi M., Nakamura M., Matsuda K., Nakamori M., Ueda K., Naka T., Ishida K., James Primus F., Yamaue H. (2007). Successful Cancer Vaccine Therapy for Carcinoembryonic Antigen (CEA)-Expressing Colon Cancer Using Genetically Modified Dendritic Cells That Express CEA and T Helper-Type 1 Cytokines in CEA Transgenic Mice. Int. J. Cancer.

[92] Gulley J.L., Arlen P.M., Tsang K.-Y., Yokokawa J., Palena C., Poole D.J., Remondo C., Cereda V., Jones J.L., Pazdur M.P. (2008). Pilot study of vaccination with recombinant CEA-MUC-1-TRICOM poxviral-based vaccines in patients with metastatic carcinoma. Clin. Cancer Res..

[93] Liu D., Guo P., McCarthy C., Wang B., Tao Y., Auguste D. (2018). Peptide Density Targets and Impedes Triple Negative Breast Cancer Metastasis. Nat. Commun..

[94] Zhang Q., Bergman J., Wiman K.G., Bykov V.J.N. (2018). Role of Thiol Reactivity for Targeting Mutant P53. Cell Chem. Biol..

[95] Bauer M.R., Joerger A.C., Fersht A.R. (2016). 2-Sulfonylpyrimidines: Mild Alkylating Agents with Anticancer Activity toward P53-Compromised Cells. Proc. Natl. Acad. Sci. USA.

[96] Synnott N.C., Bauer M.R., Madden S., Murray A., Klinger R., O’Donovan N., O’Connor D., Gallagher W.M., Crown J., Fersht A.R. (2018). Mutant P53 as a Therapeutic Target for the Treatment of Triple-Negative Breast Cancer: Preclinical Investigation with the Anti-P53 Drug, PK11007. Cancer Lett..

[97] Nijman H.W., Vermeij R., Leffers N., Van Der Burg S.H., Melief C.J., Daemen T. (2011). Immunological and Clinical Effects of Vaccines Targeting P53-Overexpressing Malignancies. J. Biomed. Biotechnol..

[98] Vijayan V., Mohapatra A., Uthaman S., Park I.K. (2019). Recent Advances in Nanovaccines Using Biomimetic Immunomodulatory Materials. Pharmaceutics.

[99] Chianese-Bullock K.A., Lewis S.T., Sherman N.E., Shannon J.D., Slingluff C.L. (2009). Multi-Peptide Vaccines Vialed as Peptide Mixtures Can Be Stable Reagents for Use in Peptide-Based Immune Therapies. Vaccine.

[100] Oka Y., Tsuboi A., Nakata J., Nishida S., Hosen N., Kumanogoh A., Oji Y., Sugiyama H. (2017). Wilms’ Tumor Gene 1 (WT1) Peptide Vaccine Therapy for Hematological Malignancies: From CTL Epitope Identification to Recent Progress in Clinical Studies Including a Cure-Oriented Strategy. Oncol. Res. Treat..

[101] Qi X.W., Zhang F., Wu H., Liu J.L., Zong B.G., Xu C., Jiang J. (2015). Wilms’ Tumor 1 (WT1) Expression and Prognosis in Solid Cancer Patients: A Systematic Review and Meta-Analysis. Sci. Rep..

[102] Coosemans A., Moerman P., Verbist G., Maes W., Neven P., Vergote I., Van Gool S.W., Amant F. (2008). Wilms’ Tumor Gene 1 (WT1) in Endometrial Carcinoma. Gynecol. Oncol..

[103] Zhang J., Guo F., Wang L., Zhao W., Zhang D., Yang H., Yu J., Niu L., Yang F., Zheng S. (2019). Screening and Identification of Non-Inflammatory Specific Protein Markers in Wilms’ Tumor Tissues. Arch. Biochem. Biophys..

[104] Goldstein N.S., Uzieblo A. (2002). WT1 Immunoreactivity in Uterine Papillary Serous Carcinomas Is Different from Ovarian Serous Carcinomas. Am. J. Clin. Pathol..

[105] Iiyama T., Udaka K., Takeda S., Takeuchi T., Adachi Y.C., Ohtsuki Y., Tsuboi A., Nakatsuka S.I., Elisseeva O.A., Oji Y. (2007). WT1 (Wilms’ Tumor 1) Peptide Immunotherapy for Renal Cell Carcinoma. Microbiol. Immunol..

[106] O’Hagan D.T., Valiante N.M. (2003). Recent Advances in the Discovery and Delivery of Vaccine Adjuvants. Nat. Rev. Drug Discov..

[107] Ghaffari-Nazari H., Tavakkol-Afshari J., Jaafari M.R., Tahaghoghi-Hajghorbani S., Masoumi E., Jalali S.A. (2015). Improving Multi-Epitope Long Peptide Vaccine Potency by Using a Strategy That Enhances CD4+ T Help in BALB/c Mice. PLoS ONE.

[108] Wu C.Y., Monie A., Pang X., Hung C.F., Wu T.C. (2010). Improving Therapeutic HPV Peptide-Based Vaccine Potency by Enhancing CD4+ T Help and Dendritic Cell Activation. J. Biomed. Sci..

[109] Zamani P., Teymouri M., Nikpoor A.R., Navashenaq J.G., Gholizadeh Z., Darban S.A., Jaafari M.R. (2020). Nanoliposomal Vaccine Containing Long Multi-Epitope Peptide E75-AE36 Pulsed PADRE-Induced Effective Immune Response in Mice TUBO Model of Breast Cancer. Eur. J. Cancer.

[110] Yazdani Z., Rafiei A., Yazdani M., Valadan R. (2020). Design an Efficient Multi-Epitope Peptide Vaccine Candidate against SARS-CoV-2: An in Silico Analysis. Infect. Drug Resist..

[111] Nezafat N., Ghasemi Y., Javadi G., Khoshnoud M.J., Omidinia E. (2014). A Novel Multi-Epitope Peptide Vaccine against Cancer: An in Silico Approach. J. Theor. Biol..

[112] Bijker M.S., van den Eeden S.J.F., Franken K.L., Melief C.J.M., van der Burg S.H., Offringa R. (2008). Superior Induction of Anti-Tumor CTL Immunity by Extended Peptide Vaccines Involves Prolonged, DC-Focused Antigen Presentation. Eur. J. Immunol..

[113] Slingluff C.L. (2011). The Present and Future of Peptide Vaccines for Cancer: Single or Multiple, Long or Short, Alone or in Combination?. Cancer J..

[114] Eskandari S., Guerin T., Toth I., Stephenson R.J. (2017). Recent Advances in Self-Assembled Peptides: Implications for Targeted Drug Delivery and Vaccine Engineering. Adv. Drug Deliv. Rev..

[115] Mohit E., Hashemi A., Allahyari M. (2014). Breast Cancer Immunotherapy: Monoclonal Antibodies and Peptide-Based Vaccines. Expert Rev. Clin. Immunol..

[116] Mufson R.A. (2006). Tumor Antigen Targets and Tumor Immunotherapy. Front. Biosci..

[117] Kawasaki T., Kawai T. (2014). Toll-like Receptor Signaling Pathways. Front. Immunol..

[118] Hos B.J., Tondini E., van Kasteren S.I., Ossendorp F. (2018). Approaches to Improve Chemically Defined Synthetic Peptide Vaccines. Front. Immunol..

[119] Bartnik A., Nirmal A.J., Yang S.-Y. (2012). Peptide Vaccine Therapy in Colorectal Cancer. Vaccines.

[120] Tsuruma T., Hata F., Furuhata T., Ohmura T., Katsuramaki T., Yamaguchi K., Kimura Y., Torigoe T., Sato N., Hirata K. (2005). Peptide-Based Vaccination for Colorectal Cancer. Expert Opin. Biol. Ther..

[121] Calvo Tardón M., Allard M., Dutoit V., Dietrich P.-Y., Walker P.R. (2019). Peptides as Cancer Vaccines. Curr. Opin. Pharmacol..

[122] Azmi F., Fuaad A.A.H.A., Skwarczynski M., Toth I. (2014). Recent Progress in Adjuvant Discovery for Peptide-Based Subunit Vaccines. Hum. Vaccin. Immunother..

[123] Chatzikleanthous D., Schmidt S.T., Buffi G., Paciello I., Cunliffe R., Carboni F., Romano M.R., O’Hagan D.T., D’Oro U., Woods S. (2020). Design of a Novel Vaccine Nanotechnology-Based Delivery System Comprising CpGODN-Protein Conjugate Anchored to Liposomes. J. Control. Release.

[124] Melssen M.M., Petroni G.R., Chianese-Bullock K.A., Wages N.A., Grosh W.W., Varhegyi N., Smolkin M.E., Smith K.T., Galeassi N.V., Deacon D.H. (2019). A Multipeptide Vaccine plus Toll-like Receptor Agonists LPS or PolyICLC in Combination with Incomplete Freund’s Adjuvant in Melanoma Patients. J. Immunother. Cancer.

[125] Yang Y., Feng R., Wang Y.Z., Sun H.W., Zou Q.M., Li H.B. (2020). Toll-like Receptors: Triggers of Regulated Cell Death and Promising Targets for Cancer Therapy. Immunol. Lett..

[126] Temizoz B., Kuroda E., Ishii K.J. (2018). Combination and Inducible Adjuvants Targeting Nucleic Acid Sensors. Curr. Opin. Pharmacol..

[127] Van Doorn E., Liu H., Huckriede A., Hak E. (2016). Safety and Tolerability Evaluation of the Use of Montanide ISATM51 as Vaccine Adjuvant: A Systematic Review. Hum. Vaccin. Immunother..

[128] Belnoue E., Di Berardino-Besson W., Gaertner H., Carboni S., Dunand-Sauthier I., Cerini F., Suso-Inderberg E.M., Wälchli S., König S., Salazar A.M. (2016). Enhancing Antitumor Immune Responses by Optimized Combinations of Cell-Penetrating Peptide-Based Vaccines and Adjuvants. Mol. Ther..

[129] Ponomarev E.D., Shriver L.P., Maresz K., Pedras-Vasconcelos J., Verthelyi D., Dittel B.N. (2007). GM-CSF Production by Autoreactive T Cells Is Required for the Activation of Microglial Cells and the Onset of Experimental Autoimmune Encephalomyelitis. J. Immunol..

[130] Yu T.W., Chueh H.Y., Tsai C.C., Lin C.T., Qiu J.T. (2016). Novel GM-CSF-Based Vaccines: One Small Step in GM-CSF Gene Optimization, One Giant Leap for Human Vaccines. Hum. Vaccin. Immunother..

[131] Bhattacharya P., Budnick I., Singh M., Thiruppathi M., Alharshawi K., Elshabrawy H., Holterman M.J., Prabhakar B.S. (2015). Dual Role of GM-CSF as a Pro-Inflammatory and a Regulatory Cytokine: Implications for Immune Therapy. J. Interferon Cytokine Res..

[132] Borriello F., Galdiero M.R., Varricchi G., Loffredo S., Spadaro G., Marone G. (2019). Innate Immune Modulation by GM-CSF and IL-3 in Health and Disease. Int. J. Mol. Sci..

[133] Zhao W., Zhao G., Wang B. (2018). Revisiting GM-CSF as an Adjuvant for Therapeutic Vaccines. Cell. Mol. Immunol..

[134] Shiomi A., Usui T. (2015). Pivotal Roles of GM-CSF in Autoimmunity and Inflammation. Mediat. Inflamm..

[135] Di Gregoli K., Johnson J.L. (2012). Role of Colony-Stimulating Factors in Atherosclerosis. Curr. Opin. Lipidol..

[136] Kim I.K., Koh C.H., Jeon I., Shin K.S., Kang T.S., Bae E.A., Seo H., Ko H.J., Kim B.S., Chung Y. (2019). GM-CSF Promotes Antitumor Immunity by Inducing Th9 Cell Responses. Cancer Immunol. Res..

[137] Decker W.K., Safdar A. (2011). Cytokine Adjuvants for Vaccine Therapy of Neoplastic and Infectious Disease. Cytokine Growth Factor Rev..

[138] Wimmers F., De Haas N., Scholzen A., Schreibelt G., Simonetti E., Eleveld M.J., Brouwers H.M.L.M., Beldhuis-Valkis M., Joosten I., De Jonge M.I. (2017). Monitoring of Dynamic Changes in Keyhole Limpet Hemocyanin (KLH)-Specific B Cells in KLHvaccinated Cancer Patients. Sci. Rep..

[139] Bi S., Bailey W., Brisson C. (2016). Performance of Keyhole Limpet Hemocyanin (KLH) as an Antigen Carrier for Protein Antigens Depends on KLH Property and Conjugation Route. J. Immunol..

[140] Aarntzen E.H.J.G., De Vries I.J.M., GöErtz J.H., Beldhuis-Valkis M., Brouwers H.M.L.M., Van De Rakt M.W.M.M., Van Der Molen R.G., Punt C.J.A., Adema G.J., Tacken P.J. (2012). Humoral Anti-KLH Responses in Cancer Patients Treated with Dendritic Cell-Based Immunotherapy Are Dictated by Different Vaccination Parameters. Cancer Immunol. Immunother..

[141] Swanson M.A., Schwartz R.S. (1967). Immunosuppressive Therapy. N. Engl. J. Med..

[142] Slovin S.F., Ragupathi G., Musselli C., Fernandez C., Diani M., Verbel D., Danishefsky S., Livingston P., Scher H.I. (2005). Thomsen-Friedenreich (TF) Antigen as a Target for Prostate Cancer Vaccine: Clinical Trial Results with TF Cluster (c)-KLH plus QS21 Conjugate Vaccine in Patients with Biochemically Relapsed Prostate Cancer. Cancer Immunol. Immunother..

[143] Razazan A., Behravan J., Arab A., Barati N., Arabi L., Gholizadeh Z., Jaafari M.R. (2017). Conjugated nanoliposome with the HER2/neu-derived peptide GP2 as an effective vaccine against breast cancer in mice xenograft model. PLoS ONE.

[144] Curry J.M., Besmer D.M., Erick T.K., Steuerwald N., Das Roy L., Grover P., Rao S., Nath S., Ferrier J.W., Reid R.W. (2019). Indomethacin Enhances Anti-Tumor Efficacy of a MUC1 Peptide Vaccine against Breast Cancer in MUC1 Transgenic Mice. PLoS ONE.

[145] Ladjemi M.Z., Jacot W., Chardès T., Pèlegrin A., Navarro-Teulon I. (2010). Anti-HER2 Vaccines: New Prospects for Breast Cancer Therapy. Cancer Immunol. Immunother..

[146] Knutson K.L., Schiffman K., Cheever M.A., Disis M.L. (2002). Immunization of Cancer Patients with a HER-2/Neu, HLA-A2 Peptide, P369-377, Results in Short-Lived Peptide-Specific Immunity. Clin. Cancer Res..

[147] Anderson B.W., Peoples G.E., Murray J.L., Gillogly M.A., Gershenson D.M., Ioannides C.G. (2000). Peptide Priming of Cytolytic Activity to HER-2 Epitope 369-377 in Healthy Individuals. Clin. Cancer Res..

[148] Ohtake J., Ohkuri T., Togashi Y., Kitamura H., Okuno K., Nishimura T. (2014). Identification of Novel Helper Epitope Peptides of Survivin Cancer-Associated Antigen Applicable to Developing Helper/Killer-Hybrid Epitope Long Peptide Cancer Vaccine. Immunol. Lett..

[149] Disis M.L., Gooley T.A., Rinn K., Davis D., Piepkorn M., Cheever M.A., Knutson K.L., Schiffman K. (2002). Generation of T-Cell Immunity to the HER-2/Neu Protein after Active Immunization with HER-2/Neu Peptide-Based Vaccines. J. Clin. Oncol..

[150] Kalli K.R., Block M.S., Kasi P.M., Erskine C.L., Hobday T.J., Dietz A., Padley D., Gustafson M.P., Shreeder B., Puglisi-Knutson D. (2018). Folate Receptor Alpha Peptide Vaccine Generates Immunity in Breast and Ovarian Cancer Patients. Clin. Cancer Res..

[151] Farran B., Pavitra E., Kasa P., Peela S., Rama Raju G.S., Nagaraju G.P. (2019). Folate-Targeted Immunotherapies: Passive and Active Strategies for Cancer. Cytokine Growth Factor Rev..

[152] Folate Receptor Alpha Peptide Vaccine with GM-CSF in Patients with Triple Negative Breast Cancer. https://clinicaltrials.gov/ct2/show/NCT02593227.

[153] MUC1 Vaccine for Triple-Negative Breast Cancer. https://www.clinicaltrials.gov/ct2/show/NCT00986609.

[154] Vaccine Therapy in Treating Patients with Breast Cancer. https://clinicaltrials.gov/ct2/show/NCT00524277.

[155] Phase Ib Trial of Two Folate Binding Protein Peptide Vaccines (E39 and J65) in Breast and Ovarian Cancer Patients (J65). https://clinicaltrials.gov/ct2/show/NCT02019524.

[156] Multipeptide Vaccine for Advanced Breast Cancer. https://clinicaltrials.gov/ct2/show/NCT00573495.

[157] Oka Y., Tsuboi A., Taguchi T., Osaki T., Kyo T., Nakajima H., Elisseeva O.A., Oji Y., Kawakami M., Ikegame K. (2004). Induction of WT1 (Wilms’ Tumor Gene)-Specific Cytotoxic T Lymphocytes by WT1 Peptide Vaccine and the Resultant Cancer Regression. Proc. Natl. Acad. Sci. USA.

[158] Oka Y., Tsuboi A., Oji Y., Kawase I., Sugiyama H. (2008). WT1 Peptide Vaccine for the Treatment of Cancer. Curr. Opin. Immunol..

[159] Holmes J.P., Benavides L.C., Gates J.D., Carmichael M.G., Hueman M.T., Mittendorf E.A., Murray J.L., Amin A., Craig D., Von Hofe E. (2008). Results of the First Phase I Clinical Trial of the Novel Ii-Key Hybrid Preventive HER-2/Neu Peptide (AE37) Vaccine. J. Clin. Oncol..

[160] Nevagi R.J., Toth I., Skwarczynski M. (2018). Peptide-Based Vaccines. Peptide Applications in Biomedicine, Biotechnology and Bioengineering.

[161] Roces C.B., Khadke S., Christensen D., Perrie Y. (2019). Scale-Independent Microfluidic Production of Cationic Liposomal Adjuvants and Development of Enhanced Lymphatic Targeting Strategies. Mol. Pharm..

[162] Salvador A., Igartua M., Hernández R.M., Pedraz J.L. (2011). An Overview on the Field of Micro- and Nanotechnologies for Synthetic Peptide-Based Vaccines. J. Drug Deliv..

[163] Ohta S., Kikuchi E., Ishijima A., Azuma T., Sakuma I., Ito T. (2020). Investigating the Optimum Size of Nanoparticles for Their Delivery into the Brain Assisted by Focused Ultrasound-Induced Blood–Brain Barrier Opening. Sci. Rep..

[164] Koerner J., Horvath D., Groettrup M. (2019). Harnessing Dendritic Cells for Poly (D,L-Lactide-Co-Glycolide) Microspheres (PLGA MS)-Mediated Anti-Tumor Therapy. Front. Immunol..

[165] Durán V., Yasar H., Becker J., Thiyagarajan D., Loretz B., Kalinke U., Lehr C.M. (2019). Preferential Uptake of Chitosan-Coated PLGA Nanoparticles by Primary Human Antigen Presenting Cells. Nanomedicine.

[166] Kroll A.V., Fang R.H., Jiang Y., Zhou J., Wei X., Yu C.L., Gao J., Luk B.T., Dehaini D., Gao W. (2017). Nanoparticulate Delivery of Cancer Cell Membrane Elicits Multiantigenic Antitumor Immunity. Adv. Mater..

[167] Gu P., Liu Z., Sun Y., Ou N., Hu Y., Liu J., Wu Y., Wang D. (2019). Angelica Sinensis Polysaccharide Encapsulated into PLGA Nanoparticles as a Vaccine Delivery and Adjuvant System for Ovalbumin to Promote Immune Responses. Int. J. Pharm..

[168] Boraschi D., Italiani P. (2015). From Antigen Delivery System to Adjuvanticy: The Board Application of Nanoparticles in Vaccinology. Vaccines.

[169] Rezvantalab S., Drude N.I., Moraveji M.K., Güvener N., Koons E.K., Shi Y., Lammers T., Kiessling F. (2018). PLGA-Based Nanoparticles in Cancer Treatment. Front. Pharmacol..

[170] Pandey A., Jain D.S. (2015). Poly Lactic-Co-Glycolic Acid (PLGA) Copolymer and Its Pharmaceutical Application. Handbook of Polymers for Pharmaceutical Technologies.

[171] Ma W., Chen M., Kaushal S., McElroy M., Zhang Y., Ozkan C., Bouvet M., Kruse C., Grotjahn D., Ichim T. (2012). PLGA Nanoparticle-Mediated Delivery of Tumor Antigenic Peptides Elicits Effective Immune Responses. Int. J. Nanomed..

[172] Chen Q., Xu L., Liang C., Wang C., Peng R., Liu Z. (2016). Photothermal Therapy with Immune-Adjuvant Nanoparticles Together with Checkpoint Blockade for Effective Cancer Immunotherapy. Nat. Commun..

[173] Chu B.Y., Al Kobiasi M., Zeng W., Mainwaring D., Jackson D.C. (2012). Chitosan-Based Particles as Biocompatible Delivery Vehicles for Peptide and Protein-Based Vaccines. Procedia Vaccinol..

[174] Singh B., Maharjan S., Sindurakar P., Cho K.H., Choi Y.J., Cho C.S. (2018). Needle-Free Immunization with Chitosan-Based Systems. Int. J. Mol. Sci..

[175] Singla A.K., Chawla M. (2010). Chitosan: Some Pharmaceutical and Biological Aspects—An Update. J. Pharm. Pharmacol..

[176] Jadidi-Niaragh F., Atyabi F., Rastegari A., Kheshtchin N., Arab S., Hassannia H., Ajami M., Mirsanei Z., Habibi S., Masoumi F. (2017). CD73 Specific SiRNA Loaded Chitosan Lactate Nanoparticles Potentiate the Antitumor Effect of a Dendritic Cell Vaccine in 4T1 Breast Cancer Bearing Mice. J. Control. Release.

[177] Pei M., Liang J., Zhang C., Wang X., Zhang C., Ma G., Sun H. (2019). Chitosan/Calcium Phosphates Nanosheet as a Vaccine Carrier for Effective Cross-Presentation of Exogenous Antigens. Carbohydr. Polym..

[178] Fang R.H., Hu C.M.J., Luk B.T., Gao W., Copp J.A., Tai Y., O’Connor D.E., Zhang L. (2014). Cancer Cell Membrane-Coated Nanoparticles for Anticancer Vaccination and Drug Delivery. Nano Lett..

[179] Harris J.C., Scully M.A., Day E.S. (2019). Cancer Cell Membrane-Coated Nanoparticles for Cancer Management. Cancers.

[180] Ruoslahti E., Bhatia S.N., Sailor M.J. (2010). Targeting of Drugs and Nanoparticles to Tumors. J. Cell Biol..

[181] Xuan M., Shao J., Li J. (2019). Cell Membrane-Covered Nanoparticles as Biomaterials. Natl. Sci. Rev..

[182] Jin J., Krishnamachary B., Barnett J.D., Chatterjee S., Chang D., Mironchik Y., Wildes F., Jaffee E.M., Nimmagadda S., Bhujwalla Z.M. (2019). Human Cancer Cell Membrane-Coated Biomimetic Nanoparticles Reduce Fibroblast-Mediated Invasion and Metastasis and Induce T-Cells. ACS Appl. Mater. Interfaces.

[183] Duan Q., Zhang H., Zheng J., Zhang L. (2020). Turning Cold into Hot: Firing up the Tumor Microenvironment. Trends Cancer.

[184] Nelde A., Rammensee H.G., Walz J.S. (2021). The Peptide Vaccine of the Future. Mol. Cell. Proteom..

[185] Vinay D.S., Ryan E.P., Pawelec G., Talib W.H., Stagg J., Elkord E., Lichtor T., Decker W.K., Whelan R.L., Kumara H.M.C.S. (2015). Immune Evasion in Cancer: Mechanistic Basis and Therapeutic Strategies. Semin. Cancer Biol..

[186] Ahmadzadeh M. (2009). OR.93. Tumor Antigen-Specific CD8 T Cells Infiltrating the Tumor Express High Levels of PD-1 and Are Functionally Impaired. Clin. Immunol..

[187] Colozza M., de Azambuja E., Personeni N., Lebrun F., Piccart M.J., Cardoso F. (2007). Achievements in Systemic Therapies in the Pregenomic Era in Metastatic Breast Cancer. Oncologist.

[188] Planes-Laine G., Rochigneux P., Bertucci F., Chrétien A.S., Viens P., Sabatier R., Gonçalves A. (2019). PD-1/PD-L1 Targeting in Breast Cancer: The First Clinical Evidences Are Emerging. a Literature Review. Cancers.

[189] Kamphorst A.O., Pillai R.N., Yang S., Nasti T.H., Akondy R.S., Wieland A., Sica G.L., Yu K., Koenig L., Patel N.T. (2017). Proliferation of PD-1+ CD8 T Cells in Peripheral Blood after PD-1-Targeted Therapy in Lung Cancer Patients. Proc. Natl. Acad. Sci. USA.

[190] Hirayama M., Nishimura Y. (2016). The Present Status and Future Prospects of Peptide-Based Cancer Vaccines. Int. Immunol..

[191] Kleponis J., Skelton R., Zheng L. (2015). Fueling the Engine and Releasing the Break: Combinational Therapy of Cancer Vaccines and Immune Checkpoint Inhibitors. Cancer Biol. Med..

